# Rad53- and Chk1-Dependent DNA Damage Response Pathways Cooperatively Promote Fungal Pathogenesis and Modulate Antifungal Drug Susceptibility

**DOI:** 10.1128/mBio.01726-18

**Published:** 2019-01-02

**Authors:** Kwang-Woo Jung, Yeonseon Lee, Eun Young Huh, Soo Chan Lee, Sangyong Lim, Yong-Sun Bahn

**Affiliations:** a Research Division for Biotechnology, Korea Atomic Energy Research Institute, Jeongeup, Republic of Korea; b Department of Biotechnology, College of Life Science and Biotechnology, Yonsei University, Seoul, Republic of Korea; c South Texas Center for Emerging Infectious Diseases (STCEID), Department of Biology, University of Texas at San Antonio, San Antonio, Texas, USA; University of British Columbia

**Keywords:** *C. neoformans*, DNA damage pathway, antifungal drug susceptibility, virulence

## Abstract

Living organisms are constantly exposed to DNA damage stress caused by endogenous and exogenous events. Eukaryotic cells have evolutionarily conserved DNA damage checkpoint surveillance systems. We previously reported that a unique transcription factor, Bdr1, whose expression is strongly induced by the protein kinase Rad53 governs DNA damage responses by controlling the expression of DNA repair genes in the basidiomycetous fungus Cryptococcus neoformans. However, the regulatory mechanism of the Rad53-dependent DNA damage signal cascade and its function in pathogenicity remain unclear. Here, we demonstrate that Rad53 is required for DNA damage response and is phosphorylated by two phosphatidylinositol 3-kinase (PI3K)-like kinases, Tel1 and Mec1, in response to DNA damage stress. Transcriptome analysis revealed that Rad53 regulates the expression of several DNA repair genes in response to gamma radiation. We found that expression of *CHK1*, another effector kinase involved in the DNA damage response, is regulated by Rad53 and that *CHK1* deletion rendered cells highly susceptible to DNA damage stress. Nevertheless, *BDR1* expression is regulated by Rad53, but not Chk1, indicating that DNA damage signal cascades mediated by Rad53 and Chk1 exhibit redundant and distinct functions. We found that perturbation of both *RAD53* and *CHK1* attenuated the virulence of C. neoformans, perhaps by promoting phagosome maturation within macrophage, reducing melanin production, and increasing susceptibility to oxidative stresses. Furthermore, deletion of both *RAD53* and *CHK1* increased susceptibility to certain antifungal drugs such as amphotericin B. This report provides insight into the regulatory mechanism of fungal DNA damage repair systems and their functional relationship with fungal virulence and antifungal drug susceptibility.

## Abstract (precis)

Genome instability is detrimental for living things because it induces genetic disorder diseases and transfers incorrect genome information to descendants. Therefore, living organisms have evolutionarily conserved signaling networks to sense and repair DNA damage. However, how the DNA damage response pathway is regulated for maintaining the genome integrity of fungal pathogens and how this contributes to their pathogenicity remain elusive. In this study, we investigated the DNA damage response pathway in the basidiomycete pathogen Cryptococcus neoformans, which causes life-threatening meningoencephalitis in immunocompromised individuals, with an average of 223,100 infections leading to 181,100 deaths reported annually. Here, we found that perturbation of Rad53- and Chk1-dependent DNA damage response pathways attenuated the virulence of C. neoformans and increased its susceptibility to certain antifungal drugs, such as amphotericin B and flucytosine. Therefore, our work paves the way to understanding the important role of human fungal DNA damage networks in pathogenesis and antifungal drug susceptibility.

## INTRODUCTION

The genome stability of cells is constantly confronted with threats imposed by both endogenous events, including DNA replication and cellular respiration generating reactive oxygen species (ROS), and exogenous stresses, including chemical and physical genotoxic DNA damage agents (1). Upon DNA damage, cells increase expression of target genes such as *RAD51* and enhance activity of effector proteins, such as Chk1, to maintain genome integrity. In humans, genetic disorders induced by DNA damage result in severe diseases, such as cancers and neurodegenerative diseases (2, 3). Therefore, surveillance and repair of damaged genomes are prerequisites for the survival and propagation of all living cells.

Living organisms contain evolutionarily conserved DNA damage checkpoint systems to counteract endogenous and exogenous DNA damage stresses (4). DNA damage response (DDR) pathways are generally composed of protein kinases, mediators, and transcription factors, which control DNA replication, DNA repair, and cell cycling (4). Among the DDR pathways, those mediated by ataxia-telangiectasia mutated (ATM) and ATM- and Rad3-related (ATR) protein kinases are evolutionarily conserved and well characterized from yeasts to humans (5). In humans, ATR and ATM kinases are members of the phosphatidylinositide-3-kinase (PI3K) family and have downstream effector kinases Chk1 and Chk2, which contain the forkhead-associated (FHA) domain and modulate DNA damage responses (6, 7). ATR kinase counteracts a wide range of DNA damage, such as double-stranded DNA breaks (DSBs) and stalled DNA replication forks, whereas ATM kinase primarily responds to DSBs (4).

In the budding yeast (Saccharomyces cerevisiae) model, DDR pathways mediated by Mec1 (human ATR homolog) and Tel1 (human ATM homolog) kinases are well characterized (8). Similarly to human effector kinases, the S. cerevisiae DNA damage response cascade activates effector kinases Chk1 and Rad53, which are the human Chk1 and Chk2 homologs, respectively (8). In S. cerevisiae, Mec1 activates both Rad53 and Chk1 via phosphorylation of their Ser/Thr phosphorylation (SQ/TQ) sites, whereas ATM and ATR kinases primarily activate Chk2 and Chk1, respectively, in humans (9–11). Upon DNA damage response, Mec1 phosphorylates the SQ/TQ consensus sites in the checkpoint adaptor protein Rad9, which recruits and binds to Rad53 through its FHA domain (12, 13). The activated Rad53 phosphorylates Dun1 kinase, which subsequently phosphorylates some transcription factors that regulate genes involved in cell cycle regulation and DNA replication and repair (14).

Recent studies revealed that the DNA repair system is also involved in antifungal drug resistance in human fungal pathogens (15–18). Perturbations in the DNA mismatch repair system, which corrects errors occurring in DNA replication and recombination (19), can increase the spontaneous mutation rate (20). In particular, deletion of *MSH2*, a homolog of Escherichia coli MutS that is one of components in the DNA mismatch repair system, has been shown to increase phenotypic diversity and adaptive antifungal drug resistance by inducing DNA mutations in Cryptococcus neoformans (17, 18). Moreover, drug-resistant Candida glabrata clinical isolates were shown to have mutations in the *MSH2* gene (21). Therefore, the acquisition of antifungal drug resistance is partly linked to genome-wide mutations governed by the DNA repair system.

C. neoformans causes fatal meningoencephalitis in immunocompromised individuals via inhalation of its dried yeasts or basidiospores through the respiratory tract and their subsequent hematogenous dissemination into the central nervous system (22, 23). The most recent epidemiological study reported that approximately 220,000 cases of cryptococcal meningitis occur globally every year and that around 80% of these lead to death (24). Recently, we showed that a novel transcription factor, Bdr1, whose expression is regulated by Rad53 modulates DNA damage responses by controlling expression levels of various DNA repair genes in C. neoformans (25). In addition, we demonstrated that components of the PI3K pathway, such as Mec1, Tel1, Chk1, and Rad53, are evolutionarily conserved and that both Mec1 and Rad53 contribute to genotoxic DNA stress response in C. neoformans (26). However, information on the regulatory mechanism of the *Cryptococcus* DNA damage repair system and its role in pathogenicity remains elusive. In this study, we investigated the regulatory mechanism of Rad53- and Chk1-dependent DNA repair systems and their roles in pathogenicity of C. neoformans. We also demonstrated that both Rad53 and Chk1 kinases promote C. neoformans virulence and modulate antifungal drug susceptibility, thereby suggesting a functional connection between DNA damage repair systems and fungal pathogenicity.

## RESULTS

### Rad53 is required for DNA damage stress response and adaptation in Cryptococcus neoformans.

**FIG 1:**
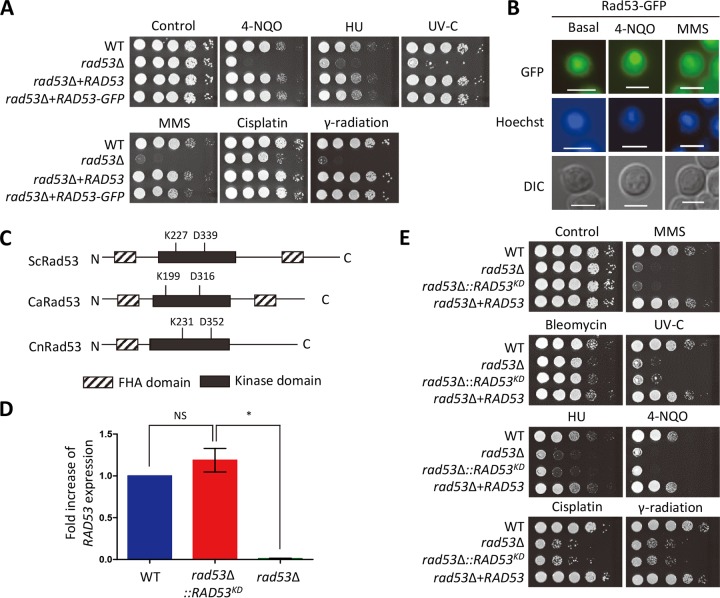
Rad53 is required for DNA damage response in C. neoformans. (A and E) C. neoformans strains (wild type [WT] H99, *rad53*Δ mutant [YSB3785], *rad53*Δ+*RAD53* mutant [KW1], *rad53*Δ+*RAD53*:*GFP* mutant [KW62], and *rad53*Δ::*RAD53^KD^* mutant [KW210]) were cultured in liquid YPD medium at 30°C overnight. The cells were 10-fold serially diluted (1 to 10^4^) and spotted on a YPD plate containing the indicated concentration of DNA damage agents (in panel A, 4-NQO, 0.1 µg/ml; HU, 100 mM; MMS, 0.03%; cisplatin, 1 mM; in panel E, 4-NQO, 0.15 µg/ml; HU, 100 mM; MMS, 0.02%; cisplatin, 1 mM; bleomycin, 0.5 µg/ml). For UV-C and gamma radiation resistance tests, the serially diluted cells were spotted on a YPD plate and then the plates were exposed to the indicated dose of UV-C (in panel A, 10 mJ/cm^2^; in panel E, 15 mJ/cm^2^) and gamma radiation (1 kGy). Cells were further incubated at 30°C and photographed daily for 1 to 3 days. (B) The cellular localization of Rad53-GFP was monitored by fluorescence microscopy. Hoechst staining was used to stain the nucleus. The scale bar represents 10 µm. DIC, differential interference contrast. (C) Comparison of catalytic active sites in S. cerevisiae Rad53 (ScRad53), C. albicans Rad53 (CaRad53), and C. neoformans Rad53 (CnRad53). (D) The expression level of *RAD53* in the *rad53*Δ::*RAD53^KD^* strain. qRT-PCR analysis was performed with cDNA synthesized from total RNA isolated from WT H99, *rad53*Δ, and *rad53*Δ::*RAD53^KD^* strains grown to the mid-log phase. Error bars indicate standard deviations. Statistical significance of difference was determined by one-way analysis of variance with Bonferroni’s multiple-comparison test (*, *P < *0.05; NS, not significant).

The *rad53*Δ mutants exhibited increased susceptibility to a series of genotoxic DNA damage insults, such as methyl methanesulfonate (MMS [an inducer of DNA alkylation]), hydroxyurea (HU [a ribonucleotide reductase inhibitor causing depletion of deoxynucleoside triphosphates {dNTPs} for DNA replication]), 4-nitroquinoline 1-oxide (4-NQO) (a DNA damage inducer through the production of reactive oxygen species), UV-C (nonionizing irradiation causing pyrimidine dimer formation), and gamma radiation (ionizing irradiation inducing diverse forms of DNA damage, such as DSBs) (Fig. 1A), which is in agreement with our previous results (25, 26). In addition, the *rad53*Δ mutant displayed increased susceptibility to cisplatin (Fig. 1A), which primarily generates intrastrand DNA cross-linking (27). Supporting these results, the *rad53*Δ+*RAD53* complemented strains exhibited wild-type (WT) levels of resistance (Fig. 1A). Therefore, Rad53 plays critical roles in response and adaptation to DNA genotoxic stress in C. neoformans.

In S. cerevisiae and humans, Rad53 and the homologous protein kinase Chk2 are localized in the nucleus (28, 29). To examine the cellular localization of Rad53 in C. neoformans, we constructed *rad53*Δ+*RAD53*-*GFP* strains (GFP, green fluorescent protein). The Rad53-GFP protein was functional because the *rad53*Δ+*RAD53*-*GFP* strain showed the WT level of resistance to DNA damage stress (Fig. 1A). Rad53 was also observed to be predominantly localized in the nucleus of C. neoformans, regardless of the presence or absence of DNA damage agents (Fig. 1B).

We next addressed whether the kinase activity of Rad53 is critical for its function. Lys (K227) and Asp (D339) residues are required for the kinase activity of S. cerevisiae Rad53 (ScRad53) (30). Similarly, mutations of corresponding K199 and D316 residues in Candida albicans Rad53 (CaRad53) abolish its function (31). Accordingly, we constructed Rad53 kinase dead mutants (*rad53*Δ::*RAD53^KD^* strain) by integrating the *RAD53* kinase dead allele (K231N and D352A; *RAD53^KD^*) into the native *RAD53* promoter locus of the *rad53*Δ mutant (Fig. 1C). The *RAD54^KD^* allele was confirmed to be expressed at WT levels (Fig. 1D). The *rad53*Δ::*RAD53^KD^* strain was as susceptible to genotoxic insults as the *rad53*Δ mutant (Fig. 1E), indicating that kinase activity is critical for Rad53 function. Taking the results together, Rad53 has an evolutionarily conserved cellular location and functions in DNA damage response and adaptation of C. neoformans through its kinase activity.

### Mec1 and Tel1 kinases phosphorylate Rad53 upon DNA damage stress.

**FIG 2:**
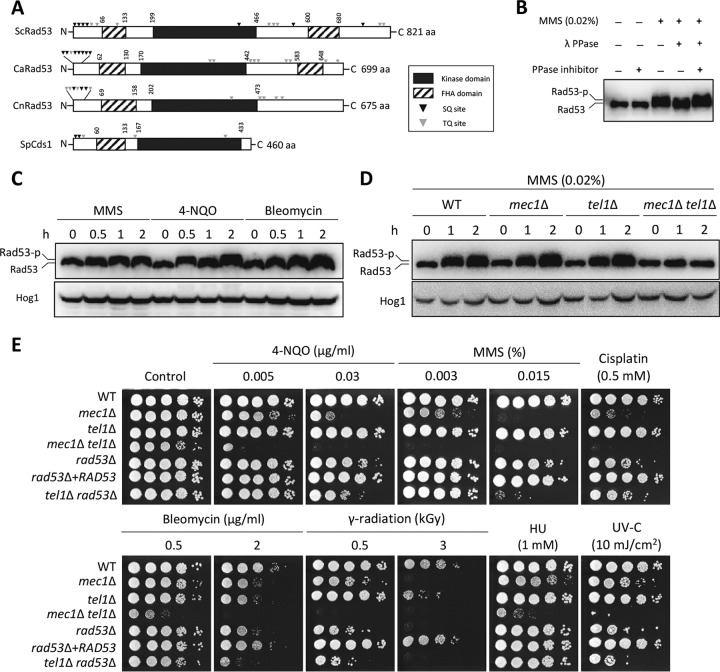
C. neoformans Rad53 is phosphorylated by both Mec1 and Tel1 kinases in response to DNA damage stress. (A) Schematic outline of Rad53 orthologs in fungi. The box with dashed lines represents the FHA domain, and the black box indicates the kinase domain. The gray inverted triangles and black inverted triangles indicate SQ and TQ sites, respectively. The domain of each protein was analyzed using Pfam (http://pfam.xfam.org/). Each protein sequence was retrieved from the genome database and NCBI [S. cerevisiae, Rad53; C. albicans, Rad53; C. neoformans, Rad53; and S. pombe, Cds1] aa, amino acids. (B and C) Phosphorylation of Rad53 was monitored by analysis of the reduced electrophoretic migration using western blotting with anti-FLAG antibody. The Rad53-4xFLAG strain was grown to the mid-logarithmic phase and then treated with MMS (0.02%) for 2 h. The cell extract was incubated at 30°C for 1 h with or without λ-phosphatase (PPase) and PPase inhibitor (B). Rad53 was phosphorylated in response to MMS (0.02%), 4-NQO (0.15 µg/ml), and bleomycin (3 µg/ml) (C). (D) Both Tel1 and Mec1 regulate Rad53 phosphorylation in response to DNA damage stress. WT Rad53-4xFLAG, *mec1*Δ Rad53-4xFLAG, *tel1*Δ Rad53-4xFLAG, and *mec1*Δ *tel1*Δ Rad53-4xFLAG strains were treated with MMS (0.02%), and then total protein was extracted from each strain for immunoblot analysis. Rad53 phosphorylation levels were monitored using anti-FLAG antibody. The same blot was stripped and then reprobed with polyclonal anti-Hog1 antibody for the loading control. (E) Mec1 and Tel1 play redundant roles in DNA damage stress response in C. neoformans. (Strains: WT Rad53-4xFLAG [YSB3806], Rad53-4xFLAG *mec1*Δ [KW102], Rad53-4xFLAG *tel1*Δ [KW104], Rad53-4xFLAG *mec1*Δ *tel1*Δ [KW449], *mec1*Δ [YSB3611], *tel1*Δ [YSB3844], *mec1*Δ *tel1*Δ [KW480], *rad53*Δ [YSB3785], *rad53*Δ+*RAD53* [KW1], and *tel1*Δ *rad53*Δ [KW106]).

FHA domains, which play essential roles in DNA damage response (32), are generally conserved in Rad53 orthologs. C. neoformans Rad53 (CnRad53) has one FHA domain near the N-terminal region, whereas ScRad53 and CaRad53 contain FHA domains at both the N- and C-terminal ends (Fig. 2A). CnRad53, like other Rad53 orthologs, also has conserved Ser/Thr phosphorylation sites (SQ/TQ) (Fig. 2A). To address whether CnRad53 undergoes phosphorylation, we constructed strains expressing *RAD53*-4xFLAG and confirmed that the Rad53-4xFLAG fusion protein was functional (see Fig. S1A in the supplemental material). The Rad53-4xFLAG protein exhibited reduced electrophoretic mobility upon treatment with MMS. However, the mobility shift was abolished by λ-phosphatase treatment, but not in the presence of both λ-phosphatase and a phosphatase inhibitor (Fig. 2B). Supporting this finding, 4-NQO and bleomycin also caused phosphorylation of Rad53 (Fig. 2C). These results suggest that CnRad53 is phosphorylated upon DNA damage stresses.

10.1128/mBio.01726-18.2 FIG S1 Construction of Rad53-4xFLAG, Chk1-4xFLAG, and Bdr1-4xFLAG strains. (A, C, and K) Diagrams for construction of the Rad53-4xFLAG, Chk1-4xFLAG, and Bdr1-4xFLAG strains. (B, D, and L) Verification of correct genotypes of the Rad53-4xFLAG, Chk1-4xFLAG, and Bdr1-4xFLAG strains. (E, F, G, H, I, and J) The correct genotype of each gene (*MEC1*, *TEL1*, or *RAD53*) deletion in the background of Rad53-4xFLAG and Chk1-4xFLAG strains was verified by Southern blot analysis using genomic DNA digested with the indicated restriction enzyme. Each membrane was hybridized with the corresponding gene-specific probe, washed, and developed. Download FIG S1, PDF file, 0.2 MB. Copyright © 2019 Jung et al. 2019 Jung et al. This content is distributed under the terms of the Creative Commons Attribution 4.0 International license.

In S. cerevisiae, Mec1 and Tel1 kinases are required for DNA damage stress and involved in Rad53 phosphorylation (33). To elucidate whether Mec1 and Tel1 are required for CnRad53 phosphorylation, we constructed *mec1*Δ, *tel1*Δ, and *mec1*Δ *tel1*Δ mutants in the Rad53-4xFLAG strain background. CnRad53 was normally phosphorylated in response to MMS in the *mec1*Δ and *tel1*Δ mutants but not in the *mec1*Δ *tel1*Δ double mutant (Fig. 2D), indicating that Tel1 and Mec1 play redundant roles in phosphorylating CnRad53 upon DNA damage response. However, the finding that the *mec1*Δ *tel1*Δ double mutant showed higher sensitivity to DNA damage stress than the *rad53*Δ mutant (Fig. 2E) indicated that CnRad53 is not the only downstream target of Mec1 and Tel1. To further elucidate the relationship between Mec1 or Tel1 and Rad53, we attempted to perform experiments using *mec1*Δ *rad53*Δ and *tel1*Δ *rad53*Δ double mutants. However, we failed to obtain the *mec1*Δ *rad53*Δ double mutant despite repeated attempts, suggesting that Rad53 and Mec1 may have a synthetic lethal relationship in C. neoformans. Supporting this conjecture, Rad53 and Mec1 are also known to have a synthetic lethal relationship in S. cerevisiae (34). Notably, the *tel1*Δ *rad53*Δ double mutant showed a greater susceptibility to 4-NQQ, bleomycin, and MMS than the *rad53*Δ mutant (Fig. 2E), suggesting that Tel1 has Rad53-independent targets. The *mec1*Δ mutant, but not the *tel1*Δ mutant, showed severe growth defects in the presence of low concentrations of DNA damage inducers. However, the *mec1*Δ *tel1*Δ double mutant showed slight growth retardation under unstressed conditions and exhibited more-severe growth defects under genotoxic stress conditions than either of the *mec1*Δ and *tel1*Δ single mutants (Fig. 2E). These data further suggest that Tel1 could contribute to DNA damage response. Collectively, the results indicate that Mec1 and Tel1 play major and minor roles, respectively, in DNA damage response and adaptation, at least in part by phosphorylation of CnRad53.

### Transcriptome profiling to explore the Rad53-dependent gene network in C. neoformans.

**FIG 3:**
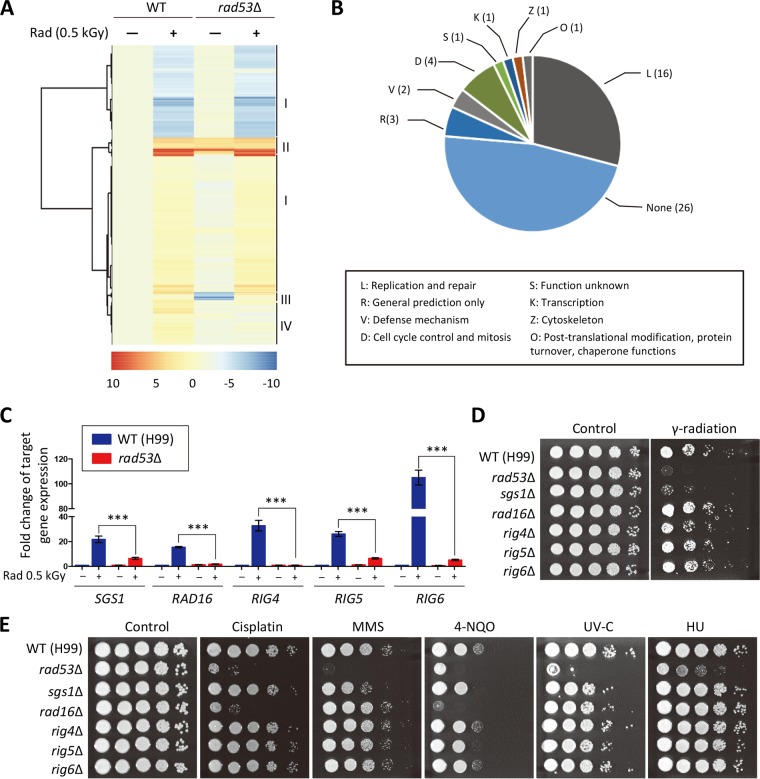
Transcriptome profiles of Rad53-dependent genes after gamma radiation exposure. (A and B) Gene clustering of Rad53-dependent genes (A) and functional categories of genes regulated by Rad53 after gamma radiation exposure determined using the EuKaryotic Orthologous Groups (KOG) of proteins (B). Numbers in parentheses represent numbers of genes in the group IV. (C) Expression levels of putative Rad53-dependent genes were verified by qRT-PCR analysis using cDNA synthesized from total RNA isolated from WT and *rad53*Δ strains with or without gamma radiation exposure. Three independent biological experiments with duplicate technical replicates were performed. Error bars indicate standard errors of the means. Statistical significance of difference was determined by one-way analysis of variance with Bonferroni’s multiple-comparison test (***, *P < *0.0001). (D and E) Characterization of roles of putative Rad53-dependent genes in gamma radiation resistance (D) and DNA damage stress resistance (E). C. neoformans strains were grown overnight at 30°C in liquid YPD medium. The 10-fold serially diluted cells (1 to 10^4^ dilutions) were spotted on a YPD plate and then exposed to the indicated doses of gamma radiation and UV-C. For DNA damage susceptibility testing, the 10-fold serially diluted cells were spotted on a YPD plate containing the indicated concentration of DNA damage insults. The plates were further incubated at 30°C and photographed daily for 1 to 3 days. (Strains: WT H99, *rad53*Δ [YSB3785], *sgs1*Δ [KW464], *rad16*Δ [KW425], *rig4*Δ [KW443], *rig5*Δ [KW385], and *rig6*Δ [KW419]).

To further elucidate the signaling circuitry downstream of CnRad53, we compared the transcriptome profiles of the WT strain and the *rad53*Δ mutants after gamma radiation exposure using RNA sequencing (RNA-seq)-based transcriptome analysis. Of a total of 6,962 genes, 5,087 and 4,768 showed statistically significantly different expression levels in the WT strain and the *rad53*Δ mutants, respectively (*P < *0.05) (see Table S1 in the supplemental material). In the WT strain, 392 genes exhibited ≥2-fold expression changes after radiation exposure (Fig. 3A; see also Table S1 in the supplemental material). Next, we categorized the radiation-induced genes into four groups based on their expression patterns. Group I included Rad53-independent radiation-regulated genes (289 genes). Group II and group III included genes exhibiting ≥2-fold-upregulated expression (33 genes) or downregulated expression (15 genes) upon deletion of *RAD53* even under basal conditions. The changes in the expression levels of these genes might have been caused by compensatory mechanisms resulting from a lack of Rad53, suggesting that Rad53 is important even under nonstress conditions. Based on the KOG categorization, most of genes in group II and group III were not functionally annotated. Group IV included radiation-regulated genes induced in the WT strain but not in the *rad53*Δ mutant (55 genes) (Fig. 3B; see also Table S1 in the supplemental material). These Rad53-dependent genes included those involved in DNA replication and repair and in cell cycle control and mitosis (Fig. 3B). Therefore, Rad53 is among the regulators that are critical for governing expression of a plethora of DNA damage repair genes in C. neoformans.

10.1128/mBio.01726-18.8 TABLE S1 The whole RNA-seq data set generated in this study. Download Table S1, XLSX file, 2.4 MB. Copyright © 2019 Jung et al. 2019 Jung et al. This content is distributed under the terms of the Creative Commons Attribution 4.0 International license.

### Identification and functional characterization of genotoxic DNA repair genes governed by Rad53.

Among the Rad53-dependent DNA damage response genes, we chose to further analyze those in the following categories: (i) evolutionarily divergent genes, such as C. neoformans 07564 (CNAG_07564), CNAG_05341, and CNAG_03906, and (ii) evolutionarily conserved but hitherto-uncharacterized C. neoformans genes. The latter included CNAG_02512 and CNAG_03654, which are highly orthologous to S. cerevisiae Rad16 and Sgs1, respectively. First, we verified our transcriptome data for the five selected genes using quantitative reverse transcription-PCR (qRT-PCR) analysis. Consistent with the transcriptome data, all these genes were significantly induced in the WT strain but not in the *rad53*Δ mutant after gamma radiation exposure (Fig. 3C). Given that we assigned the name “*RIG*” (radiation-induced gene) to genes induced after radiation exposure in our previous study (25), we designated CNAG_05341, CNAG_07564, and CNAG_03906 *RIG4*, *RIG5*, and *RIG6*, respectively.

To elucidate the function of the five Rad53-dependent genes, we generated *rig4*Δ, *rig5*Δ, *rig6*Δ, *rad16*Δ, and *sgs1*Δ mutants. To exclude any unlinked mutational effect and to validate a mutant phenotype, we constructed two independent mutants for each gene (see Fig. S2 in the supplemental material). The two independent mutants for each gene were phenotypically identical (data not shown). The *sgs1*Δ mutants exhibited increased susceptibility to gamma radiation, whereas the *rig4*Δ, *rig5*Δ, and *rig6*Δ mutants showed WT levels of gamma radiation resistance (Fig. 3D). Notably, the *rad16*Δ mutant was also as resistant to gamma radiation as the WT (Fig. 3D), which is in stark contrast to a previous report that the *rad16*Δ mutant showed increased gamma radiation sensitivity in S. cerevisiae (35). Next, we further examined whether these genes are involved in other genotoxic stress responses. The *sgs1*Δ mutant showed a growth defect in response to 4-NQO and MMS (Fig. 3E). However, deletion of *RIG4*, *RIG5*, or *RIG6* did not lead to growth defects under DNA damage stress conditions (Fig. 3E). In contrast to the radiation sensitivity phenotype, the *rad16*Δ mutants exhibited increased susceptibility to 4-NQO and cisplatin (Fig. 3E). Taken together, our data imply that evolutionarily conserved DNA damage response regulators might be functionally divergent among fungi.

10.1128/mBio.01726-18.3 FIG S2 Construction of Rad53-dependent gene mutants, including *chk1*Δ and *rad53*Δ single mutants and *rad53*Δ *chk1*Δ, *tel1*Δ *rad53*Δ, and *mec1*Δ *tel1*Δ double mutants. (A, C, E, G, I, K, M, O, and Q) Diagram for construction of the mutants. (B, D, F, H, J, L, N, P, and R) The correct genotype of each deletion mutant was verified by Southern blot analysis using genomic DNA digested with the indicated restriction enzyme. Each membrane was hybridized with the corresponding gene-specific probe, washed, and developed. Download FIG S2, PDF file, 0.2 MB. Copyright © 2019 Jung et al. 2019 Jung et al. This content is distributed under the terms of the Creative Commons Attribution 4.0 International license.

### Rad53 and Chk1 play redundant and discrete roles in genotoxic stress in C. neoformans.

**FIG 4:**
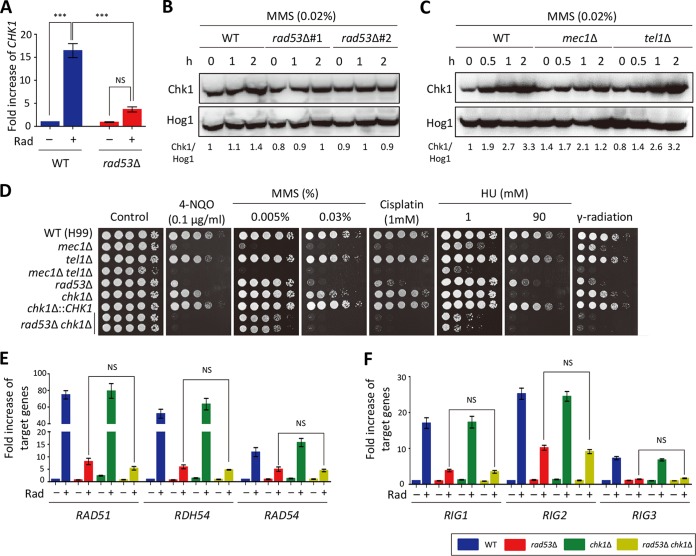
Rad53 and Chk1 play redundant and discrete roles in DNA damage response of C. neoformans. (A) Rad53 regulates *CHK1* expression after gamma radiation exposure. Quantitative RT-PCR analysis was performed using cDNA synthesized from total RNA isolated from WT H99 and *rad53*Δ mutant strains with or without gamma radiation exposure. Three independent biological experiments with duplicate technical replicates were performed. Error bars indicate standard errors of the means. Statistical significance of difference was determined by one-way analysis of variance with Bonferroni’s multiple-comparison test (***, *P < *0.0001; NS, not significant). (B and C) Protein levels of Chk1 upon DNA damage stress. WT Chk1-4xFLAG, Chk1-4xFLAG *rad53*Δ, Chk1-4xFLAG *tel1*Δ, and Chk1-4xFLAG *mec1*Δ strains were exposed to MMS (0.02%), and then total protein was extracted from each strain for immunoblot analysis. The Chk1 protein levels were monitored by immunoblot analysis with anti-FLAG antibody. The same blot was stripped and then reprobed with polyclonal anti-Hog1 antibody for the loading control. The relative abundance of the Chk1 protein was quantitatively measured by calculating band intensity of Chk1 and Hog1 proteins. (D) Each C. neoformans strain was cultured in liquid YPD medium overnight at 30°C, serially diluted (1 to 10^4^), and then spotted on a YPD plate containing the indicated concentration of DNA damage agents. For gamma radiation resistance test, the serially diluted strains spotted on the solid YPD medium were exposed to gamma radiation (0.5 kGy). Cells were further incubated for 1 to 3 days and photographed daily. (E and F) Quantitative RT-PCR analysis was performed using cDNA synthesized from total RNA of WT H99 and *rad53*Δ, *chk1*Δ, and *rad53*Δ *chk1*Δ mutants with or without gamma radiation exposure. Three independent biological experiments with duplicate technical replicates were performed. Error bars indicate standard errors of the means. Statistical significance of difference was determined by one-way analysis of variance with Bonferroni’s multiple-comparison test (NS, not significant). (Strains: WT [H99], *mec1*Δ [YSB3611], *tel1*Δ [YSB3844], *mec1*Δ *tel1*Δ [KW480], *rad53*Δ [YSB3785], *chk1*Δ [KW191], *chk1*Δ+*CHK1* [KW355], *rad53*Δ *chk1*Δ [KW250 and KW251], Chk1-4xFLAG [KW69], Chk1-4xFLAG *rad53*Δ [KW408 and KW409], Chk1-4xFLAG *tel1*Δ [KW161], and Chk1-4xFLAG *mec1*Δ [KW170]).

One of notable findings in the transcriptome analysis described above was that the *CHK1* expression was strongly induced in the WT strain but not in the *rad53*Δ mutant. We confirmed this finding using qRT-PCR analysis (Fig. 4A). Similarly, *CHK1* expression was also strongly induced by MMS in the WT strain but not in the *rad53*Δ mutant (see Fig. S3A in the supplemental material). To determine if Chk1 protein levels are also controlled by Rad53, we constructed strain Chk1-4xFLAG (see Fig. S1C in the supplemental material). Chk1 protein levels also increased upon MMS treatment but not in the *rad53*Δ mutant (Fig. 4B). To examine whether the Chk1 protein level is controlled by Tel1 and Mec1, we deleted *TEL1* or *MEC1* genes in the Chk1-4xFLAG strains. Deletion of *MEC1*, but not *TEL1*, decreased the level of Chk1 induction caused by MMS treatment (Fig. 4C). Given that mRNA transcript and protein levels of *CHK1* were positively regulated by Rad53, we hypothesized that *CHK1* overexpression could at least partially overcome the resistance of the *rad53*Δ mutant to DNA damage stress. However, *CHK1* overexpression did not increase the DNA damage resistance in the *rad53*Δ mutant (see Fig. S3G in the supplemental material), suggesting that coinduction of all or some of the remaining Rad53-dependent genes is required for restoration of DNA damage resistance in the *rad53*Δ mutant.

10.1128/mBio.01726-18.4 FIG S3 Constitutive overexpression of *CHK1* did not rescue the resistance of the *rad53*Δ mutant to DNA damage stress. (A) Expression levels of *CHK1* in WT and *rad53*Δ mutant strains in response to MMS. qRT-PCR analysis was performed using cDNA synthesized from total RNA isolated from WT strain H99 and the *rad53*Δ mutant with or without treatment with MMS (0.02%). (B) Diagram for construction of *CHK1* overexpression strains in the WT strain and the *rad53*Δ mutants. (C and E) The correct genotype of each deletion mutant was verified by Southern blot analysis using genomic DNA digested with the indicated restriction enzyme. Each membrane was hybridized with *CHK1*-specific probe, washed, and developed. (D and F) Quantitative measurement of *CHK1* gene expression. (G) Phenotypic assay of *CHK1* overexpression strains (WT strain [H99], *chk1*Δ mutant [KW191], *CHK1oe* strain [KW388 and KW399], *rad53*Δ mutant [YSB3785], and *CHK1oe rad53*Δ mutant [KW395 and KW396]) in response to DNA damage stress. (H) Phosphorylation of Chk1 was monitored through electrophoretic migration using anti-FLAG antibody. Cells containing Chk1-4xFLAG were grown to the mid-logarithmic phase and then exposed to MMS (0.02%) for 2 h. The cell extract was incubated at 30°C for 1 h with or without λ-phosphatase and phosphatase inhibitor. Download FIG S3, PDF file, 0.2 MB. Copyright © 2019 Jung et al. 2019 Jung et al. This content is distributed under the terms of the Creative Commons Attribution 4.0 International license.

The previous finding that Chk1 is phosphorylated by the Mec1 and Tel1 upstream kinases in S. cerevisiae (36, 37) and our data showing that *CHK1* overexpression did not restore DNA damage resistance in the *rad53*Δ mutant in C. neoformans led us to examine whether Chk1 undergoes phosphorylation in response to DNA damage stress in C. neoformans. In contrast to the results seen with Rad53, the electrophoretic mobility of Chk1 was not changed upon MMS treatment (see Fig. S3H in the supplemental material). This result indicates that Chk1 might not be phosphorylated or that the number of phosphorylated sites in Chk1 was not sufficiently abundant for detection by the electrophoretic mobility shift change.

As *CHK1* expression and protein production were regulated by Rad53 in C. neoformans, we constructed the *chk1*Δ mutant and its complemented strain to determine the function of Chk1 in the DNA damage stress response. The *chk1*Δ mutant was more susceptible to 4-NQO and MMS than the WT and the corresponding complemented strain (Fig. 4D). However, the *chk1*Δ mutant did not display increased susceptibility to cisplatin (Fig. 4D). Notably, although the *chk1*Δ mutant was generally less susceptible to DNA damage stresses than the *rad53*Δ mutant, the *chk1*Δ mutant exhibited a higher level of susceptibility to HU than the *rad53*Δ mutant (Fig. 4D). To unravel the redundant roles of Rad53 and Chk1, we constructed *rad53*Δ *chk1*Δ double mutants. Remarkably, the *rad53*Δ *chk1*Δ double mutants were much more susceptible to MMS, HU, 4-NQO, cisplatin, and gamma radiation than either single mutant (Fig. 4D). All these data suggest that Rad53 and Chk1 play redundant but discrete roles in genotoxic DNA stress response and adaptation.

Our transcriptome data revealed that a number of Bdr1-dependent radiation-responsive genes, including *RAD51*, *RAD54*, *RDH54*, *RIG1*, *RIG2*, and *RIG3* (25), were still inducible in the *rad53*Δ mutant, albeit to a much less extent than in the WT strain. Given that Chk1 and Rad53 played redundant roles in the genotoxic stress response, we quantitatively analyzed the expression patterns of *RAD51*, *RAD54*, *RDH54*, *RIG1*, *RIG2*, and *RIG3* in the *rad53*Δ, *chk1*Δ, and *rad53*Δ *chk1*Δ mutants compared with the WT strain with or without gamma radiation exposure. Unexpectedly, we found that the gamma radiation-mediated induction levels of these genes in the *rad53*Δ *chk1*Δ double mutant were similar to those in the *rad53*Δ mutant (Fig. 4E and F). This result implies that Chk1 controls the DNA damage response in a Rad53-independent manner.

### Bdr1 controls expression levels of genes involved in DNA repair system as a downstream factor of Rad53 but not Chk1.

**FIG 5:**
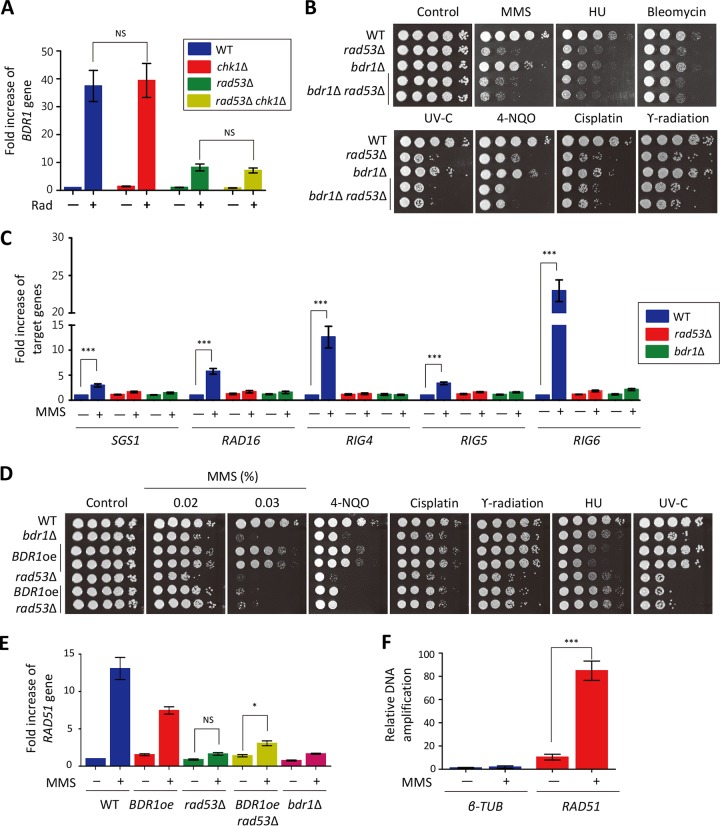
Bdr1 is a bona-fide transcription factor downstream of Rad53 in C. neoformans. (A) Expression levels of *BDR1* in the WT strain and *rad53*Δ, *chk1*Δ, and *rad53*Δ *chk1*Δ mutants. Quantitative RT-PCR analysis was performed using cDNA synthesized from total RNA of WT H99, *rad53*Δ, *chk1*Δ, and *rad53*Δ *chk1*Δ mutants with or without gamma radiation exposure. Error bars indicate standard errors of the means. (NS, not significant). (B and D) C. neoformans strains (WT [H99], *rad53*Δ [YSB3785], *bdr1*Δ [KW137], *rad53*Δ *bdr1*Δ [KW190 and KW238], *BDR1*oe [KW198 and KW199], and *BDR1*oe *rad53*Δ [KW242 and KW243]) were grown in liquid YPD medium overnight at 30°C, serially diluted (1 to 10^4^), and then spotted on YPD plates containing the indicated concentration of DNA damage agents (in panel B, MMS [0.015%], HU [90 mM], bleomycin [2 µg/ml], 4-NQO [0.075 µg/ml], and cisplatin [1 mM]; in panel D, HU [90 mM], 4-NQO [0.075 µg/ml], and cisplatin [1 mM]). For UV-C and gamma radiation resistance tests, the serially diluted strains spotted on the solid YPD medium were exposed to UV (10 mJ/cm^2^ [C] or 12.5 mJ/cm^2^ [C]) or gamma radiation (0.5 kGy). (C) Expression levels of Rad53-dependent genes in the WT strain and the *rad53*Δ and *bdr1*Δ mutants. The qRT-PCR analysis was performed using cDNA synthesized from total RNA of WT H99 and the *rad53*Δ and *bdr1*Δ mutants with or without MMS treatment. Error bars indicate standard errors of the means. (***, *P < *0.0001). (E) Overexpression of *BDR1* induced expression of *RAD51* upon MMS treatment. Quantitative RT-PCR analysis was performed using cDNA synthesized from total RNA of WT H99, *BDR1oe* (KW198), *rad53*Δ (YSB3785), *BDR1*oe *rad53*Δ (KW242), and *bdr1*Δ (KW137) with or without MMS treatment (1 h). Error bars indicate standard errors of the means. Asterisks indicate statistical significance of difference (*, *P < *0.05; NS, not significant). (F) ChIP-qPCR was performed using the Bdr1-4xFLAG strain with three biological replicates. β-Tubulin was used as the negative control. Error bars indicate standard errors of the means. (***, *P < *0.0001). Three independent biological experiments with duplicate technical replicates were performed in experiments whose results are presented in this figure. Statistical significance of difference was determined by one-way analysis of variance with Bonferroni’s multiple-comparison test.

We previously reported that *BDR1* expression is controlled by Rad53 after gamma radiation exposure but that its induction is not completely abolished in the *rad53*Δ mutant (25), indicating that an upstream factor other than Rad53 controls *BDR1* expression during the DNA damage response. To test whether Chk1 might be one of the genes responsible for regulation of Bdr1, we measured *BDR1* induction after gamma radiation exposure in the *chk1*Δ and *rad53*Δ *chk1*Δ mutants. The *BDR1* induction levels were equivalent in the *rad53*Δ and *rad53*Δ *chk1*Δ mutants (Fig. 5A), indicating that *BDR1* expression is regulated by Rad53 but not by Chk1. These data further confirm that Chk1 and Rad53 have separate functions.

To further demonstrate that Bdr1 lies downstream of Rad53, we constructed two independent *rad53*Δ *bdr1*Δ double mutants. The *rad53*Δ *bdr1*Δ double mutants were as susceptible to all DNA damage agents as the *rad53*Δ mutant (Fig. 5B), indicating that Bdr1 is a transcription factor downstream of Rad53. To further support this finding, we monitored MMS-mediated induction of 19 of the 54 Rad53-dependent genes, including *SGS1*, *RAD16*, *RIG4*, *RIG5*, and *RIG6*, in the *bdr1*Δ mutant (Fig. 5C and Fig. S4). In agreement with the RNA-seq data, expression of all of the 19 genes was significantly induced by MMS treatment in the WT strain, but their induction was much reduced to similar degrees in the *bdr1*Δ and *rad53*Δ mutants. These results indicate that Bdr1 controls the majority of Rad53-dependent genes during DNA damage stress. Given that *BDR1* expression is mainly controlled by Rad53, we hypothesized that *BDR1* overexpression may restore the resistance of the *rad53*Δ mutant to DNA damage stresses. To test this, we generated constitutive *BDR1* overexpression strains in the *rad53*Δ mutant background (see Fig. S5 in the supplemental material). *BDR1* overexpression partially restored the resistance of the *rad53*Δ mutant to MMS, 4-NQO, cisplatin, and gamma radiation but not to HU and UV-C (Fig. 5D). Supporting this, we found that *BDR1* overexpression also weakly restored *RAD51* induction in the *rad53*Δ mutant (Fig. 5E). To demonstrate that Bdr1 directly binds to the *RAD51* promoter, we performed chromatin immunoprecipitation (ChIP)-qPCR analysis. We found that direct binding of Bdr1 to the *RAD51* promoter was strongly induced by MMS treatment (Fig. 5F). Taking the results together, Bdr1 is a bona-fide transcription factor downstream of Rad53 and regulates a subset of Rad53-dependent genes for DNA damage response and adaptation.

10.1128/mBio.01726-18.5 FIG S4 Bdr1 controls expression levels of a majority of Rad53-dependent genes. Total RNA was isolated from cells treated with MMS (0.02%) for 1 h or left untreated. The qRT analysis was performed with gene-specific primers using cDNA synthesized from the total RNA. Duplicate technical experiments with three biological samples were performed. Statistical significance was determined using Bonferroni’s multiple-comparison test. Error bars indicate standard errors of the means. Asterisks indicate the statistical significance of differences in the fold change of target genes (***, *P < *0.001). Download FIG S4, PDF file, 0.1 MB. Copyright © 2019 Jung et al. 2019 Jung et al. This content is distributed under the terms of the Creative Commons Attribution 4.0 International license.

10.1128/mBio.01726-18.6 FIG S5 Construction of *BDR1* overexpression strains in the *rad53*Δ mutant. (A) Diagram for construction of the *BDR1* constitutive overexpression strain. (B) The correct genotype of each deletion mutant was verified by Southern blot analysis using genomic DNA digested with the indicated restriction enzyme. The membrane was hybridized with *BDR1*-specific probe, washed, and developed. (C) Quantitative measurement of *BDR1* gene expression in the *rad53*Δ mutant. Download FIG S5, PDF file, 0.1 MB. Copyright © 2019 Jung et al. 2019 Jung et al. This content is distributed under the terms of the Creative Commons Attribution 4.0 International license.

### Rad53 and Chk1 cooperatively regulate virulence in C. neoformans.

**FIG 6:**
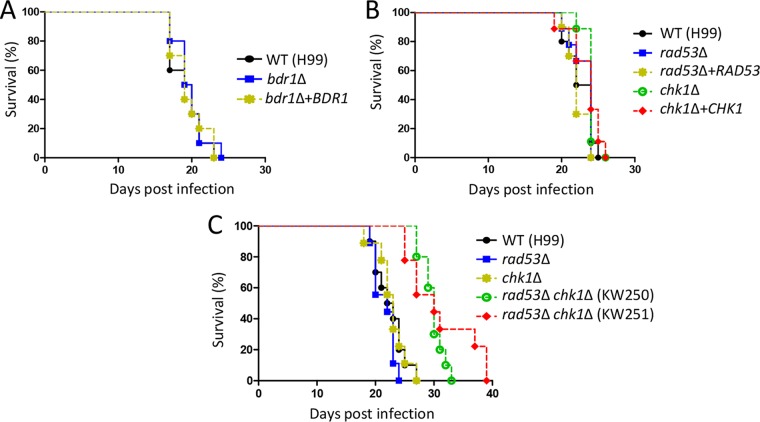
Rad53 and Chk1 promote the virulence of C. neoformans. A/Jcr mice were infected with 5 × 10^5^ *Cryptococcus* cells (in panel A, WT H99, *bdr1*Δ [KW137], and *bdr1*Δ+*BDR1* [KW193]; in panel B, WT H99, *rad53*Δ [YSB3785], *rad53*Δ+*RAD53* [KW1], *chk1*Δ [KW191], and *chk1*Δ+*CHK1* [KW355]; in panel C, WT H99, *rad53*Δ [YSB3785], *chk1*Δ [KW191], and *rad53*Δ *chk1*Δ [KW250 and KW251]) by intranasal instillation. Survival was monitored for 39 days postinfection. The survival curve was statistically analyzed by log rank (Mantel-Cox) test. *P > *0.05 for WT H99 versus strain *bdr1*Δ (A); *P > *0.05 for WT H99 versus strain *rad53*Δ and WT H99 versus strain *chk1*Δ (B); *P > *0.05 for WT H99 versus strain *rad53*Δ and WT H99 versus strain *chk1*Δ, *P < *0.0001 for WT H99 versus strain *rad53*Δ *chk1*Δ (KW250), and *P = *0.0002 for WT H99 versus strain *rad53*Δ *chk1*Δ (KW251) (C).

We next addressed the role of the DNA damage pathway in the virulence of C. neoformans using a murine model of systemic cryptococcosis. Before we conducted the *in vivo* virulence assay, we confirmed that *rad53*Δ, *chk1*Δ, and *rad53*Δ *chk1*Δ mutants exhibited WT levels of growth at a host physiological temperature (37°C) (see Fig. S6A in the supplemental material). We found that the survival rate of mice infected with the *bdr1*Δ mutant strain was indistinguishable from that of mice infected with the WT strain or the *bdr1*Δ+*BDR1* strain (Fig. 6A). Mice infected with the *rad53*Δ or *chk1*Δ mutant also showed a survival rate similar to that of mice infected with WT strain and the corresponding complemented strains (Fig. 6B). Given that Rad53 and Chk1 play redundant and discrete roles in DNA damage response and adaptation, we monitored the survival rate of mice infected with the *rad53*Δ *chk1*Δ double mutant strain compared with that of mice infected with each single mutant. Notably, *rad53*Δ *chk1*Δ double mutants exhibited attenuated virulence compared with the WT strain and the *rad53*Δ and *chk1*Δ single mutants (Fig. 6C). These data indicate that Rad53 and Chk1 cooperatively regulate virulence of C. neoformans.

10.1128/mBio.01726-18.7 FIG S6 The role of the DNA damage response pathways in virulence factor production. (A) Each strain was incubated in liquid YPD medium overnight at 30°C. Cells were 10-fold serially diluted (1 to 10^4^) and then spotted (3 µl) on YPD plates. The plates were incubated at 30°C or 37°C for 1 to 3 days. Cells were photographed daily. (B) Qualitative assay of capsule production in DNA damage response pathway mutants. Strains were cultured in solid-agar-based DMEM at 37°C for 2 days. After incubation, cells were stained by the use of India ink and observed under a microscope (Olympus BX51). (C) Each C. neoformans strain was grown at 30°C overnight and spotted onto solid Niger seed medium containing the indicated concentration of glucose. Cells were further incubated at 30°C for 1 to 2 days and photographed with a digital camera. Download FIG S6, PDF file, 0.3 MB. Copyright © 2019 Jung et al. 2019 Jung et al. This content is distributed under the terms of the Creative Commons Attribution 4.0 International license.

### Chk1 is involved in phagosome maturation within macrophage phagocytizing C. neoformans.

**FIG 7:**
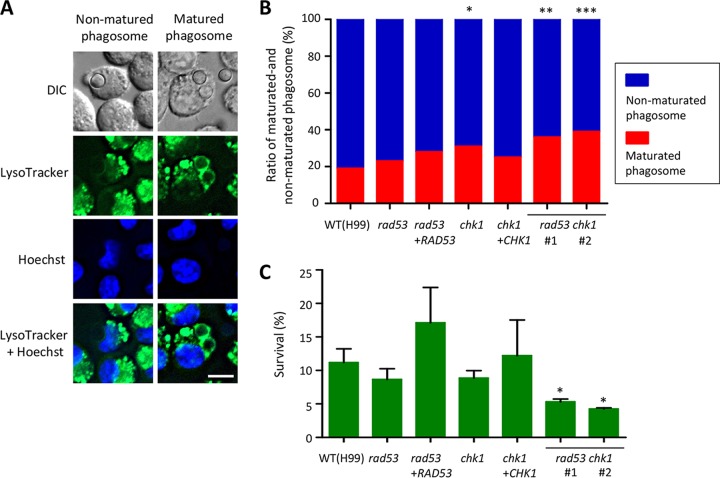
Chk1 inhibits phagosome maturation during phagocytosis of C. neoformans by macrophages. (A) C. neoformans WT H99 can block phagosome maturation upon phagocytosis by macrophages. In this experiment, approximately 80% macrophages containing H99 did not undergo maturation (left panels) based on LysoTracker staining for acidification of matured phagosomes. The rest of phagosomes (∼20%) exhibited LysoTracker-positive signaling, suggesting that phagosome maturation had occurred (right panels). This difference in the patterns of LysoTracker signaling on phagosomes with *Cryptococcus* was used to determine phagosome maturation with mutants *rad53*Δ, *chk1*Δ, and *rad53*Δ *chk1*Δ along with the WT strain. Scale bar = 10 µm. Numerical aperture = 0.75. (B) Phagosomes containing the *chk1*Δ and *rad35*Δ *chk1*Δ mutants showed a higher ratio of maturation than those containing WT. (*, *P* = 0.034; **, *P* = 0.017; ***, *P* = 0.0053.) (C) The *rad53*Δ *chk1*Δ mutants exhibit a lower ratio of survival than the WT strain (*, *P < *0.05) during interactions with macrophages.

C. neoformans modulates the milieu of the phagosome environment such that phagosome maturation or phagolysosome formation is blocked (38). Destruction of pathogens within phagosomes by phagocytic cells such as macrophages is a basic innate immunity mechanism of mammalian hosts, blockade of which is therefore one of virulence attributes of this fungus. Based on the roles of Rad53 and Chk1 in the virulence of C. neoformans, we examined whether mutation of *RAD53* and/or *CHK1* would result in an alteration of this capacity of the fungus. The WT strain and the *rad53*Δ, *chk1*Δ, and *rad53*Δ *chk1*Δ double mutants were cocultured with the J774A.1 macrophage cell line. To monitor the acidification of phagosomes, LysoTracker Green DND-26 was added to the culture media. Under conditions of phagosome maturation, phagosomes with *Cryptococcus* turn green, and a green ring signal of LysoTracker is observable (left panel in Fig. 7A); on the other hand, if phagosome maturation is blocked or arrested, no green ring signal around *Cryptococcus* cells can be observed in the phagosomes (right panel in Fig. 7A). Phagosomes containing the WT strain exhibited a low overall level of phagosome maturation (81% nonmatured phagosomes versus 19% matured phagosomes, *n* = 85) (Fig. 7B). Lack of *RAD53* did not appear to affect the phagosome blockade attribute, where 23% of phagosomes with the *rad53*Δ mutants underwent phagosome maturation, a result not significantly different from that seen with the WT strain (*n* = 49, *P = *0.69). Phagosomes containing the *chk1*Δ mutants, however, exhibited a higher maturation ratio (31% of cells, *n* = 64, *P = *0.034) than was seen with the WT strain. Phagosomes containing each *rad53*Δ *chk1*Δ double mutant also exhibited higher maturation ratios than were seen with the WT strain (for KW250, 36%, *n* = 50, *P = *0.017; for KW251, 39%, *n* = 86, *P = *0.0053); however, the differences from the results seen with the *chk1*Δ single mutant were not significant (*P = *0.47 for KW250 and *P = *0.25 for KW251). In congruence with the phagosome maturation rate, the survival rates of each *rad53*Δ *chk1*Δ double mutant seen during interactions with macrophages were significantly lowered (*P = *0.0485 or *P = *0.081). These observations indicate that the virulence cooperatively regulated by Rad53 and Chk1 is associated with *Cryptococcus* blocking phagosome maturation and increasing survival during interactions with phagocytic cells.

### Rad53 and Chk1 play redundant roles in melanin biosynthesis and the oxidative stress response in C. neoformans.

**FIG 8:**
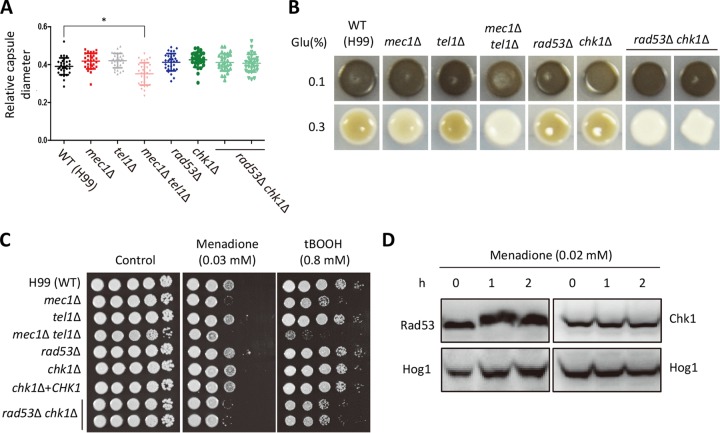
Rad53 and Chk1 cooperatively regulate melanin production and oxidative stress in C. neoformans. (A) Quantitative measurement of capsule production. Strains grown overnight at 30°C in liquid YPD medium were spotted in solid-agar-based DMEM, further incubated at 37°C for 2 days, scrapped, stained by India ink, and observed under a microscope (Olympus BX51). Relative capsule diameters were calculated as described in Materials and Methods and shown in the scatter plot. A total of 30 cells in each strain were measured for capsule production. For statistical analysis, three biologically independent experiments were performed. Statistical significance of difference was determined by one-way analysis of variance with Bonferroni’s multiple-comparison test. Asterisks indicate statistical significance of difference (*, *P < *0.05). (B) Each C. neoformans strain was grown at 30°C overnight and spotted onto solid l-DOPA medium containing 0.1% or 0.3% glucose. Cells were further incubated at 30°C and photographed using a digital camera 1 to 2 days postincubation. Glu, glucose. (C) Each strain (WT [H99], *mec1*Δ [YSB3611], *tel1*Δ [YSB3844], *mec1*Δ *tel1*Δ [KW480], *rad53*Δ [YSB3785], *chk1*Δ [KW191], *chk1*Δ+*CHK1* [KW355], and *rad53*Δ *chk1*Δ [KW250 and KW251]) was grown overnight at 30°C in liquid YPD medium, 10-fold serially diluted (1 to 10^4^), and then spotted (3 µl) on YPD plates containing the indicated concentration of oxidative stress inducers. Cells were further incubated at 30°C for 1 to 3 days and photographed daily. (D) WT Rad53-4xFLAG and Chk1-4xFLAG strains were treated with 0.02 mM menadione, and then total protein was extracted for immunoblot analysis. Rad53 phosphorylation and Chk1 protein levels were monitored in the separated gels using anti-FLAG antibody. The same blot was stripped and reprobed with polyclonal anti-Hog1 antibody for the loading control.

To counteract the deleterious effects of the host immune system, C. neoformans produces various virulence factors, including capsule and melanin (39, 40). The fact that mice infected with *rad53*Δ *chk1*Δ double mutant strains showed attenuated virulence and higher phagosome maturation than each single mutant led us to examine whether DNA damage response effector kinases Rad53 and Chk1 are involved in the virulence factor production of C. neoformans. We found that the *mec1*Δ *tel1*Δ double mutant produced lower levels of capsule. However, the *rad53*Δ *chk1*Δ double mutants produced WT levels of capsule in both quantitative and qualitative capsule assays (Fig. 8A; see also Fig. S6B in the supplemental material). In contrast, *rad53*Δ *chk1*Δ double mutants exhibited reduced melanin production in both Niger seed and l-3,4-dihydroxyphenylalanine (l-DOPA) media. Particularly under high (0.3%)-glucose conditions, the *mec1*Δ *tel1*Δ and *rad53*Δ *chk1*Δ double mutants produced less melanin than either single mutant (Fig. 8B; see also Fig. S6C in the supplemental material). These data indicate that Rad53 and Chk1 play redundant roles in melanin production in C. neoformans.

As melanin displays antioxidant activity, we tested whether Rad53 and Chk1 are also involved in oxidative stress responses without the involvement of melanin. The *rad53*Δ *chk1*Δ double mutants were more sensitive to menadione, which is a superoxide generator, and to *tert*-butyl hydroperoxide, which is an alkyl peroxide, than the WT strain (Fig. 8C). These results led us to monitor Rad53 phosphorylation and increases in Chk1 production during oxidative stress. Rad53 underwent phosphorylation in response to menadione, but the levels of Chk1 proteins did not change (Fig. 8D), indicating that Rad53 may play a role in oxidative stress responses. Taking the results together, Rad53 and Chk1 contribute to C. neoformans virulence by controlling melanin production and oxidative stress resistance during host infection.

### The DNA damage pathway is involved in antifungal drug resistance.

**FIG 9:**
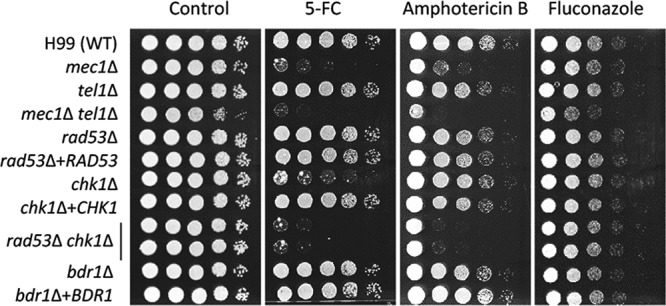
Rad53 and Chk1 play redundant roles in antifungal drug susceptibility. Each C. neoformans strain was cultured in liquid YPD medium at 30°C, 10-fold serially diluted (1 to 10^4^), spotted (3 µl) on YPD plate containing the indicated concentration of antifungal drugs (5-FC, 200 µg/ml; amphotericin B, 0.8 µg/ml; fluconazole, 16 µg/ml), and further incubated at 30°C for 1 to 3 days. Cells were photographed daily.

Amphotericin B (AmpB) and azoles are used for treatment of cryptococcosis as initial and maintenance therapeutic options, respectively (41). Particularly for AmpB, combination therapy with flucytosine is highly recommended for initial anticryptococcal therapy (42, 43). We hypothesized that Rad53- and Chk1-dependent pathways could be involved in flucytosine resistance because flucytosine inhibits DNA synthesis. Supporting that hypothesis, the *mec1*Δ *tel1*Δ double mutant was more susceptible to flucytosine than the WT strain and each single mutant (Fig. 9). Notably, the *chk1*Δ mutant, but not the *rad53*Δ mutant, exhibited increased susceptibility to flucytosine, indicating that Chk1 plays a major role in flucytosine resistance. However, as the *rad53*Δ *chk1*Δ double mutant was even more susceptible to flucytosine than the *chk1*Δ mutant (Fig. 9), Rad53 likely plays a minor role in flucytosine resistance. Similarly to flucytosine, the *mec1*Δ *tel1*Δ double mutant was more susceptible to AmpB than the WT strain and each single mutant (Fig. 9). Similarly, the *rad53*Δ *chk1*Δ mutant was more susceptible to AmpB than the WT strain and each single mutant. The *bdr1*Δ mutant was as resistant to AmpB as the WT strain (Fig. 9). In contrast to flucytosine and AmpB, the DNA damage pathway did not appear to be involved in azole drug resistance (Fig. 9). Taking the results together, the Rad53- and Chk1-dependent DNA damage pathways are involved in flucytosine and AmpB resistance of C. neoformans.

## DISCUSSION

**FIG 10:**
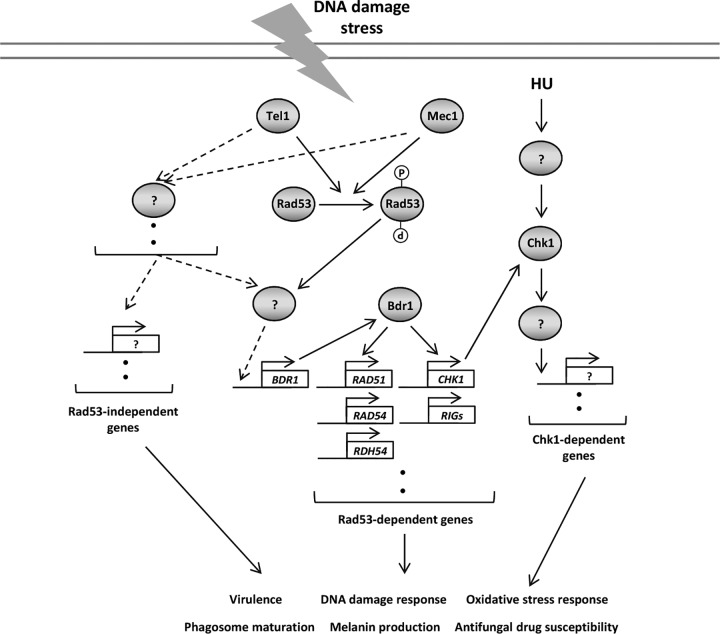
The proposed model of Rad53- and Chk1-dependent DNA damage response pathways in C. neoformans. C. neoformans has evolutionarily conserved and distinct DNA damage response pathways to counteract deleterious effects caused by DNA damage stresses. In response to exogenous or endogenous DNA damage, Mec1 and Tel1 kinases cooperatively phosphorylate Rad53 kinase. The activated Rad53 induces the expression of its downstream genes involved in DNA damage repair system, including Chk1 kinase, by inducing the expression of Bdr1 transcription factor. Chk1, which is another kinase downstream of PI3K, is mainly induced by the Mec1-Rad53 signaling pathway in response to DNA damage stress. Rad53 and Chk1 play both redundant and discrete roles in DNA damage response and adaptation, oxidative stress response, melanin production, antifungal drug susceptibility, phagosome maturation, and virulence in C. neoformans.

In this study, we elucidated for the first time the complex regulatory mechanism of Rad53- and Chk1-dependent DNA damage response pathways in C. neoformans and the corresponding biological function in fungal pathogenicity and antifungal drug resistance (Fig. 10). Here we demonstrated that Rad53 and Chk1 play redundant and distinct roles in genotoxic stress. Transcriptome analysis revealed that Rad53 governs expression of a plethora of DNA damage repair genes. Most importantly, we demonstrated that Rad53 and Chk1 cooperatively control virulence of C. neoformans by modulating phagosome maturation upon phagocytosis by macrophage, melanin production, and oxidative stress responses. Furthermore, Rad53- and Chk1-dependent pathways are involved in antifungal drug susceptibility.

The DNA damage response pathway mediated by PI3K-like kinases is well conserved among fungal pathogens. Nevertheless, the functions of individual components appear to be divergent among fungi. In C. albicans, strains deleted of *MEC1* or *RAD53* genes exhibit increased susceptibility to DNA damaging agents (44, 45). However, a *CHK1* homolog (orf19.3751) appears to be essential for viability (Candida Genome Database). Although Ustilago maydis has Atr1 and Atm1 orthologs, Atr1 is nonessential and controls phosphorylation of Chk1 upon DNA damage stress, whereas Atm1 is essential for growth (46). Notably, in contrast to other fungal pathogens, U. maydis has only the Chk1 kinase, which plays critical roles in DNA damage response, cell cycle regulation, and virulence, and not the Chk2/Rad53-like kinase (47, 48). However, the fact that the *atr1*Δ mutant is more susceptible to DNA damage stress than the *chk1*Δ mutant indicates that another kinase(s) downstream of Atr1 counteracts DNA damage stress along with Chk1 in U. maydis. In contrast, the DNA damage response pathway of Fusarium graminearum is similar to that of C. neoformans. The PI3K-like kinases and their downstream kinases are not essential for viability, and the level of HU sensitivity in these F. graminearum mutants is similar to that in the corresponding C. neoformans mutants (49). Furthermore, *atr1*Δ *atm1*Δ and *chk1*Δ *chk2*Δ double mutants exhibited greater susceptibility to HU than any single mutant, as in C. neoformans. Taking the results together, the regulatory mechanisms of DNA damage response pathways in diverse fungal pathogens exhibit common and distinct features.

The unique finding in this study was that Chk1 induction is regulated by Rad53 in C. neoformans. Phosphorylation sites in other Rad53 orthologs, such as Cds1 in S. pombe and Chk2 in mammals, were previously identified (50–53). In S. pombe, threonine 11 in Cds1 is required for phosphorylation under conditions of HU treatment, whereas threonine 68 is required for radiation-induced phosphorylation (51, 53). Moreover, mutation of threonine 8 partially decreased Cds1 phosphorylation in response to HU and the *cds1*^T11A^ strain and the *cds1*Δ mutant showed different levels of sensitivity to HU, suggesting that the phosphorylation sites in Rad53 orthologs are redundant and distinct in response to DNA damage stress. Similarly, in mammals, ATM and ATR have overlapping and distinct phosphorylation sites in Chk2. Similarly to S. pombe Cds1, the Chk2 phosphorylation sites depend on DNA damage stress (52). Therefore, because Rad53 was not phosphorylated in the *mec1*Δ *tel1*Δ double mutant, C. neoformans Rad53 may also have Mec1-specific, Tel1-specific, and Mec1- and Tel1-redundant phosphorylation sites. Moreover, given that Chk1 induction in the *tel1*Δ mutant was similar to that in the WT strain, Mec1-dependent phosphorylation sites in Rad53 appear to be required for Chk1 induction. Therefore, Mec1- and/or Tel1-dependent phosphorylation sites in C. neoformans Rad53 and their responses to diverse DNA damage stress inducers need to be identified and characterized in future studies.

The most notable feature of the DNA damage response pathway in C. neoformans is the presence of the Bdr1 transcription factor, which is structurally and functionally divergent from other DNA damage-related transcription factors, such as Rfx1. The Rfx transcription factor is evolutionarily conserved as a regular factor X (RFX) DNA binding domain and has been well characterized in the eukaryotic kingdom (54, 55). The fungal Rfx1 orthologs are widely found in the ascomycetes fungi and are known to regulate DNA damage responses, cell cycle regulation, and yeast-hyphal growth (54, 56–58). However, C. neoformans does not have a transcription factor harboring an RFX domain. Instead, it is likely that the Bdr1 bZIP-domain transcription factor could be its functional ortholog in controlling DNA damage response (25). Nevertheless, the Bdr1-like transcription factor is not likely a conserved ortholog throughout the basidiomycetes species, because U. maydis has one *RFX1* ortholog but no Bdr1 ortholog. In addition to the structural differences between Bdr1 and Rfx1 orthologs, their regulatory mechanisms also appear to be distinct. In S. cerevisiae, Crt1 is phosphorylated by Dun1 kinase and is then dissociated from the DNA binding sites with an Ssn6-Tup1 corepressor complex upon DNA damage stress (59). In contrast, Bdr1 appears to be transcriptionally regulated because its basal level is very low under unstressed conditions but its expression is rapidly induced by DNA damage stresses. Supporting this, here we demonstrate that *BDR1* overexpression could partly restore the DNA damage resistance in the *rad53*Δ mutant and induce the expression of DNA damage response target genes, such as *RAD51*. Furthermore, because Crt1 acts as corepressor with the Ssn6-Tup1 complex, deletion of *CRT1* confers DNA damage resistance in S. cerevisiae (59). Similarly, mutation of *RFX2*, which encodes an Rfx1 paralog in C. albicans, increases UV irradiation resistance (57). The findings that Bdr1 positively regulates the expression of many DNA repair genes and that deletion of *BDR1* results in growth defects under conditions of DNA damage stress strongly indicate that Bdr1 promotes the DNA damage response and adaptation in C. neoformans, which is in stark contrast to other RFX-type transcription factors involved in fungal DNA damage responses.

Here we demonstrated that both the Rad53-dependent and Chk1-dependent pathways contribute to the pathogenicity of C. neoformans. Although the role of DNA damage response pathways in fungal pathogenicity has been also reported in other fungal pathogens, their modes of action appear to be different. In C. albicans, a dimorphic transition between yeast and hyphal forms in response to external signals is one of critical virulence factors (60). Interestingly, genotoxic stress induces a morphogenetic change from the yeast form to the filamentous form (45). Deletion of *RFX2* causes hyperfilamentous growth and thereby attenuates virulence in C. albicans (57). Similarly to C. albicans, morphogenetic changes such as production of infectious dikaryotic hypha are regulated by the cell cycle in U. maydis (61). The impaired cell cycle arrest caused by deletion of *CHK1* or *ATR1* leads to inappropriate formation of infective filament, which results in attenuated virulence (46, 48). Upon infection through the respiratory tract, C. neoformans encounters alveolar macrophages and is internalized through opsonic and nonopsonic phagocytosis. After internalization of cells, the phagosome, which is a single-membraned vesicle that contains C. neoformans, undergoes maturation. During this process, C. neoformans is exposed to a harsh antimicrobial environment including such elements as the respiratory burst (oxidative burst) and addition of antimicrobial degrading enzymes (62). Consequently, inherent resistance against phagocytosis and phagosome maturation is an indispensable virulence attribute for C. neoformans. Interestingly, our data suggest that Chk1 is in part required for the survival of *Cryptococcus* inside macrophages, since mutation in *CHK1* enhanced phagosome maturation. Similarly, we demonstrated that the *rad53*Δ *chk1*Δ mutants showed a lower survival rate than the WT strain within the macrophage. In addition, we found that antioxidant effects such as melanin production mediated by the DNA damage pathway rather than DNA damage repair *per se* appeared to be critical for virulence in C. neoformans. Supporting this idea, *rad53*Δ, *chk1*Δ, and *bdr1*Δ mutants, which were not defective in melanin production, did not have attenuated virulence even though they exhibited severe growth defects in response to exogenous genotoxic stress. At this point, however, we cannot not exclude the possibility that proper DNA damage response and adaptation also contribute to the virulence of C. neoformans. We previously reported that the DNA damage response pathway could also be involved in morphogenetic transition of C. neoformans (26). *MEC1* and *TEL1* deletions enhance and reduce mating efficiency in C. neoformans, respectively, while *RAD53* deletion does not alter the mating response. These data imply that signaling components in the DNA damage pathway may independently contribute to morphogenetic transition of C. neoformans. In *Cryptococcus*, morphogenetic transition involving cell size change, such as titan cell formation, appears to be more important for virulence than transition involving cell shape change (63). A previous study revealed that strains with a deletion of *PCL103*, which encodes the cell cycle regulator G1 cyclin E, produce more titan cells (64). Therefore, the relationships among morphogenetic transition, DNA damage response pathways, and cell cycle regulation and its contribution to *Cryptococcus* pathogenicity should be further explored in future studies.

Finally, we demonstrated that the DNA damage response pathway affects antifungal drug resistance in C. neoformans. Recent increases in systemic and invasive mycoses and the emergence of antifungal-drug-resistant strains have become critical issues for public health, owing to the limited availability of antifungal drugs and their toxic side effects. Therefore, identification of novel antifungal drug targets is urgently needed. Our study data suggest that perturbation of the DNA damage response pathway may increase susceptibility to flucytosine and AmpB, the combination of which is widely used for initial anticryptococcal treatment. Chemogenomic analysis using an S. cerevisiae knockout library has revealed that diverse cellular processes, including nitrogen metabolism, cell cycle, and DNA repair, are involved in flucytosine resistance (65). It is thus likely that impaired DNA damage responses would increase the antifungal effect exerted by flucytosine. Our results showed that the Chk1-mediated pathway plays more critical roles in flucytosine resistance than the Rad53-mediated pathway. As the Rad53-dependent Bdr1 transcription factor is dispensable for flucytosine resistance, a Chk1-dependent transcription factor(s) should be responsible for such activity and needs to be identified and characterized in the near future. A recent study reported that AmpB treatment increased cellular ROS levels in S. cerevisiae and C. albicans, whereas treatment with fluconazole or flucytosine did not generate ROS (66). Supporting this, deletion of both *RAD53* and *CHK1* did not have an influence on resistance to cell membrane stresses exerted by SDS or fludioxonil, which are not involved in ROS production (data not shown). Therefore, we can speculate that increased ROS levels may further damage chromosomal DNA and that the effect can be further aggravated by impairment of the DNA damage response pathway. As Chk1 may not be directly used as an antifungal drug target due to its structural conservation, identification of another downstream or upstream target(s) of the Chk1-dependent DNA damage response pathway could be useful to develop novel antifungal drugs, particularly for combination therapy with AmpB and/or flucytosine.

## MATERIALS AND METHODS

More details of the materials and methods employed are provided in Text S1 in the supplemental material.

10.1128/mBio.01726-18.1 TEXT S1 Supplemental methods. Download Text S1, DOCX file, 0.03 MB. Copyright © 2019 Jung et al. 2019 Jung et al. This content is distributed under the terms of the Creative Commons Attribution 4.0 International license.

### Ethics statement.

All animal experiments performed in this study were approved by the Committee on the Use and Care of Animals at the Republic of Korea Atomic Energy Research Institute (KAERI-IACUC-2017-018).

### Strains and growth conditions.

The C. neoformans strains used in this study are described in Table S2 in the supplemental material. Cells were cultured and maintained in YPD (yeast extract-peptone-dextrose) medium unless stated otherwise.

10.1128/mBio.01726-18.9 TABLE S2 Strains used in this study. Download Table S2, DOCX file, 0.03 MB. Copyright © 2019 Jung et al. 2019 Jung et al. This content is distributed under the terms of the Creative Commons Attribution 4.0 International license.

### Construction of *Cryptococcus* mutant strains.

Genetic information for each gene was obtained from FungiDB (http://fungidb.org/fungidb/). Each gene deletion mutant was constructed in the C. neoformans serotype A H99S (WT) strain background using split marker/double-joint PCR (DJ-PCR) strategies (67, 68). All primers used in this study are listed in Table S3. In the first-round PCR, the 5′- and 3′-flanking regions of each gene were amplified using primer pairs L1/L2 and R1/R2, respectively, and H99 genomic DNA as the template. The nourseothricin acetyltransferase (*NAT*) selection marker was amplified using primers M13Fe and M13Re. In the second-round PCR, a fusion fragment that contains the 5′-flanking regions of the target gene and the *NAT* selection marker was amplified using primers L1/B1455. Another fusion fragment that harbors the 3′-flanking regions of the target gene and the *NAT* selection marker was amplified using primers R2/B1454. Then, the two DJ-PCR products were combined, precipitated onto 600 µg of gold microcarrier beads, and introduced into the H99S strain by biolistic transformation as previously described (67). To construct *rad53*Δ *chk1*Δ, *tel1*Δ *rad53*Δ, and *mec1*Δ *tel1*Δ double mutants, *CHK1*, *RAD53*, and *MEC1* gene disruption cassettes were similarly generated using DJ-PCR with primers listed in Table S3. A *CHK1*::*NEO* disruption cassette was introduced into the *rad53*Δ (YSB3785) mutant and *RAD53*::*NEO* and *MEC1*::*NEO* disruption cassettes were introduced into the *tel1*Δ (YSB3844) mutant by biolistic transformation. Stable transformants selected on YPD medium containing nourseothricin or G418 were initially screened by diagnostic PCR, and their correct genotype was confirmed using Southern blot analysis as previously described (69).

10.1128/mBio.01726-18.10 TABLE S3 Primers used in this study. Download Table S3, DOCX file, 0.03 MB. Copyright © 2019 Jung et al. 2019 Jung et al. This content is distributed under the terms of the Creative Commons Attribution 4.0 International license.

### Western blot analysis.

Each 4xFLAG tagging strain was grown in liquid YPD medium for 16 h at 30°C and subcultured in fresh liquid YPD medium at 30°C until the optical density at 600 nm (OD_600_) of the culture medium reached approximately 0.8. A portion of the cell culture was collected for the zero-time sample, and the remaining culture was treated with DNA damage agents such as MMS (0.02%), 4-NQO (0.15 µg/ml), and bleomycin (3 µg/ml) for the indicated amount of time. To monitor Rad53 phosphorylation levels, a primary mouse anti-FLAG antibody (F3165; Sigma) and a secondary anti-mouse IgG horseradish peroxidase-conjugated antibody (SC-2013; Santa Cruz Biotechnology) were used. To monitor Hog1 protein levels as the loading control, a primary rabbit polyclonal Hog1 antibody (SC-9079; Santa Cruz Biotechnology) and a secondary anti-rabbit IgG horseradish peroxidase-conjugated antibody (A6154; Sigma) were used. The membrane was developed using an ECL system (ChemiDoc Imaging system; Bio-Rad). For λ-phosphatase assay, the Rad53-4xFLAG strain was grown in YPD medium at 30°C overnight and subcultured into 200 ml fresh YPD medium with an inoculum at an OD_600_ of 0.2 and further incubated for 5 h at 30°C until the OD_600_ reached approximately 0.8. Next, 100 ml of the culture was collected for the zero-time control and the remaining culture was treated with MMS (final concentration, 0.02%) and further incubated for 2 h at 30°C. The collected cells were divided into two halves. One half was disrupted in lysis buffer (50 mM Tris-HCl [pH 7.5], 150 mM NaCl, 0.5 mM EDTA, and 0.5% Triton X-100 supplemented with protease inhibitor cocktail [Invitrogen] and phenylmethylsulfonyl fluoride [PMSF]) with phosphatase inhibitor cocktail (Sigma), and the other half was disrupted in the same lysis buffer without phosphatase inhibitor cocktail (Sigma) using a bead beater (Precellys) for 6 cycles (30 s homogenization with 3 min in ice). The protein extracted from each sample was incubated with PMP buffer (50 mM HEPES [pH 7.5], 100 mM NaCl, 2 mM dithiothreitol [DTT], 0.01% Brij 35, 1 mM MnCl_2_) and 800 units of λ-phosphatase (New England BioLabs) for 1 h at 30°C.

### RNA-seq and data analysis.

Poly(A) mRNA was purified using oligo(dT) magnetic beads (Qiagen, Germany) from total RNA isolated as described above and was disrupted into short fragments. Double-stranded cDNA fragments were synthesized with sequencing adaptors using a TruSeq stranded mRNA prep kit (Illumina). Next, the library was subjected to paired-end sequencing (2 × 150 bp) using an Illumina NextSeq500 platform. The raw reads were first preprocessed by trimming the adaptors and short (less than 36 bp) sequences, and duplicates and ambiguous nucleotides were eliminated using Trimmomatic (70). Reads from individual samples were aligned to the C. neoformans genome sequence using Bowtie with default parameters (71). Transcript abundances quantified in reads per kilobase per million (RPKM) were estimated using RNA-seq by expectation maximization (RSEM) (72). The threshold of expression was set to 0.3 RPKM, and genes mapped with fewer than five reads were also eliminated (73). To identify the differential expression patterns of transcripts, the trimmed mean of M-values (TMM)-normalized fragments per kilobase per million (FPKM) {calculated as total exon fragments/[mapped reads (millions) × exon length (in kilobases)]} matrix was used for generating heatmaps in the R programming environment. The read counts calculated by RSEM were used for the identification of differentially expressed genes (DEGs) using EdgeR (74). DEG identification data were set as 2-fold changes with a false-decrease rate of <0.01. The analyses were done using in-house scripts and R packages.

### Virulence assay.

For infection, each strain (WT [H99], *rad53*Δ [YSB3785], *rad53*Δ+*RAD53* [KW1], *chk1*Δ [KW191], *chk1*Δ+*CHK1* [KW355], *rad53*Δ *chk1*Δ [KW250 and KW251], *bdr1*Δ [KW137], and *bdr1*Δ+*BDR1* [KW193]) was cultured in YPD liquid medium at 30°C for 16 h. After incubation, cells were pelleted by centrifugation and washed three times in phosphate-buffered saline (PBS). Cells were adjusted to 10^7^ cells/ml in sterile PBS. A/Jcr female mice (7 weeks of age; 9 or 10 mice per group) anesthetized by intraperitoneal injection of 2,2,2-tribromoethanol (Avertin) were infected via intranasal instillation with 50 µl of cells (5 × 10^5^ cells). Mice were daily checked for signs of morbidity (extension of cerebral portion of the cranium, abnormal gait, paralysis, seizures, convulsion, or coma) and their body weight. Animals exhibiting signs of morbidity or weight loss were sacrificed by administration of CO_2_. The log rank (Mantel-Cox) test was used for analyzing statistical differences between survival curves and calculating *P* values.

### Phagosome maturation assay.

J774 A.1 macrophage cell lines were cultured and maintained in Dulbecco’s modified Eagle’s medium (DMEM) (Gibco) containing 10% fetal bovine serum (Invitrogen) at 37°C in a 5% CO_2_ environment. The macrophage cells (1 × 10^6^/ml) were subcultured and placed in a glass-bottom 24-well plate at 24 h before fungal challenges. The medium was replaced with fresh medium containing *Cryptococcus* cells (1 × 10^6^/ml) and LysoTracker Green DND-26 (0.6 μM) and Hoechst 33342 (800 ng/ml). After 30 min, maturation of phagosomes containing *Cryptococcus* cells was monitored by using a Zeiss Axio Observer D1 system with filters for enhanced green fluorescent protein (eGFP)/fluorescein isothiocyanate (FITC)/Alexa 488 and DAPI (4′,6-diamidino-2-phenylindole)/Hoechst (Zeiss). Phagosomes containing *Cryptococcus* cells were randomly selected, and the levels of matured and nonmatured phagosomes were determined by LysoTracker signal analysis. To measure the survival rates of each strain during interactions with macrophages, opsonized *Cryptococcus* cells were cocultured with J774A.1 macrophages at a multiplicity of infection (MOI) of 20:1 for 24 h at 37°C in a 5% CO_2_ environment. After centrifugation at 3,500 rpm for 5 min, media were replaced with double-distilled water (ddH_2_O) to lyse macrophage cells for 5 min. The obtained *Cryptococcus* cells were diluted and spread onto YPD agar, and CFU counting was performed. As a control, each *Cryptococcus* strain was incubated in the tissue culture media without macrophages for 24 h at 37°C in a 5% CO_2_ environment and CFU levels were measured, which served as a reference to calculate the survival rate. The significance of differences in the ratios of matured phagosomes to nonmatured phagosomes and in the rates of survival of mutants and WT cells was determined by Bonferroni’s multiple-comparison tests. Before coculture with macrophages, all *Cryptococcus* cells were opsonized with human serum or anti-GXM antibody for 1 h at 37°C.

### Data availability.

The RNA-seq data generated by this study are available at Gene Expression Omnibus (GEO accession no. GSE117227). We will provide any materials used in this study upon request.
