# Supplementary figures and images for: Rad53- and Chk1-Dependent DNA Damage Response Pathways Cooperatively Promote Fungal Pathogenesis and Modulate Antifungal Drug Susceptibility
Source: mBio. 2019 Jan 2;10(1):e01726-18. doi: 10.1128/mBio.01726-18 (PMC6315099; doi:10.1128/mBio.01726-18)

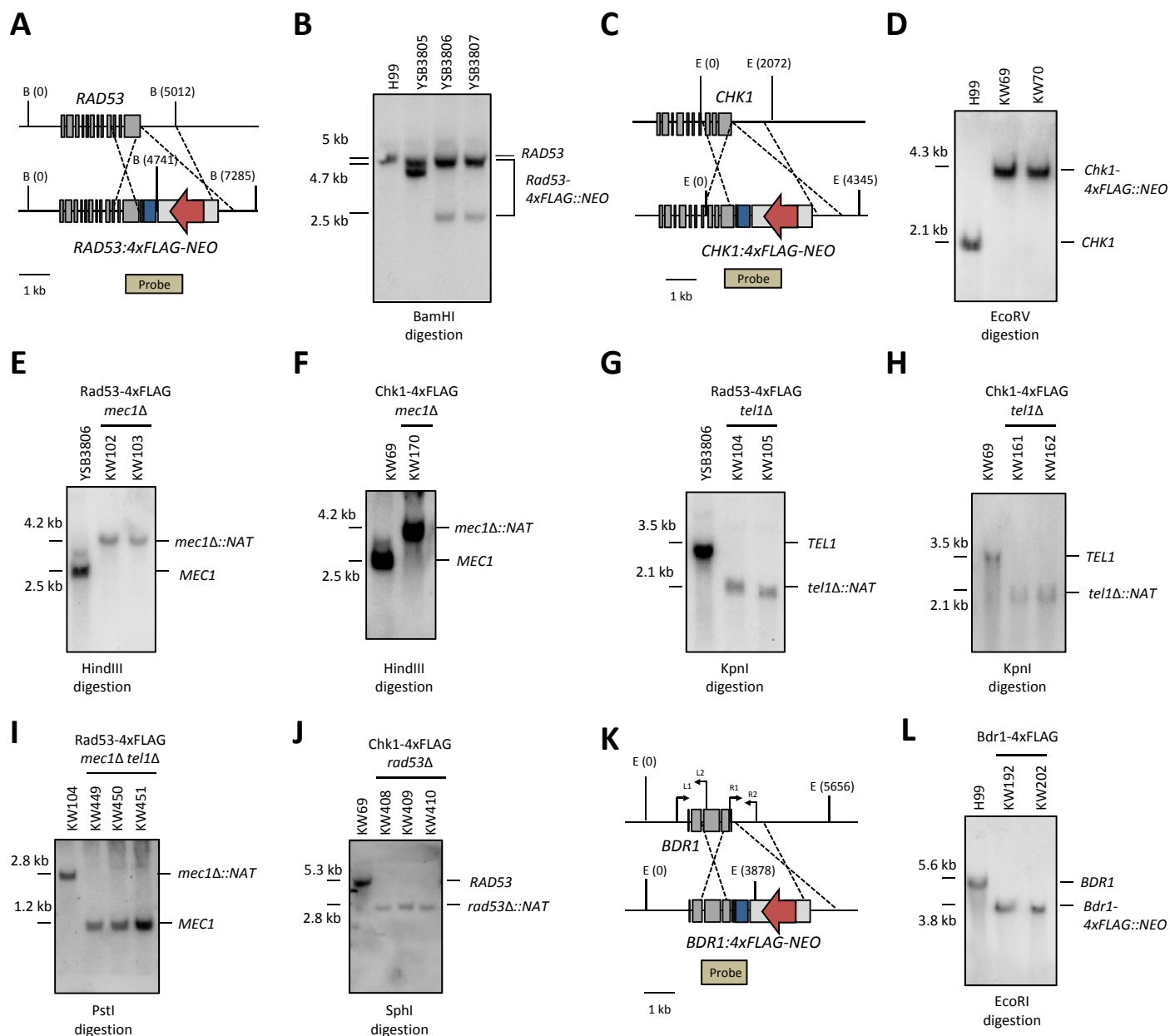

Supplement: FIG S1 [file mbo004184242sf1.pdf]

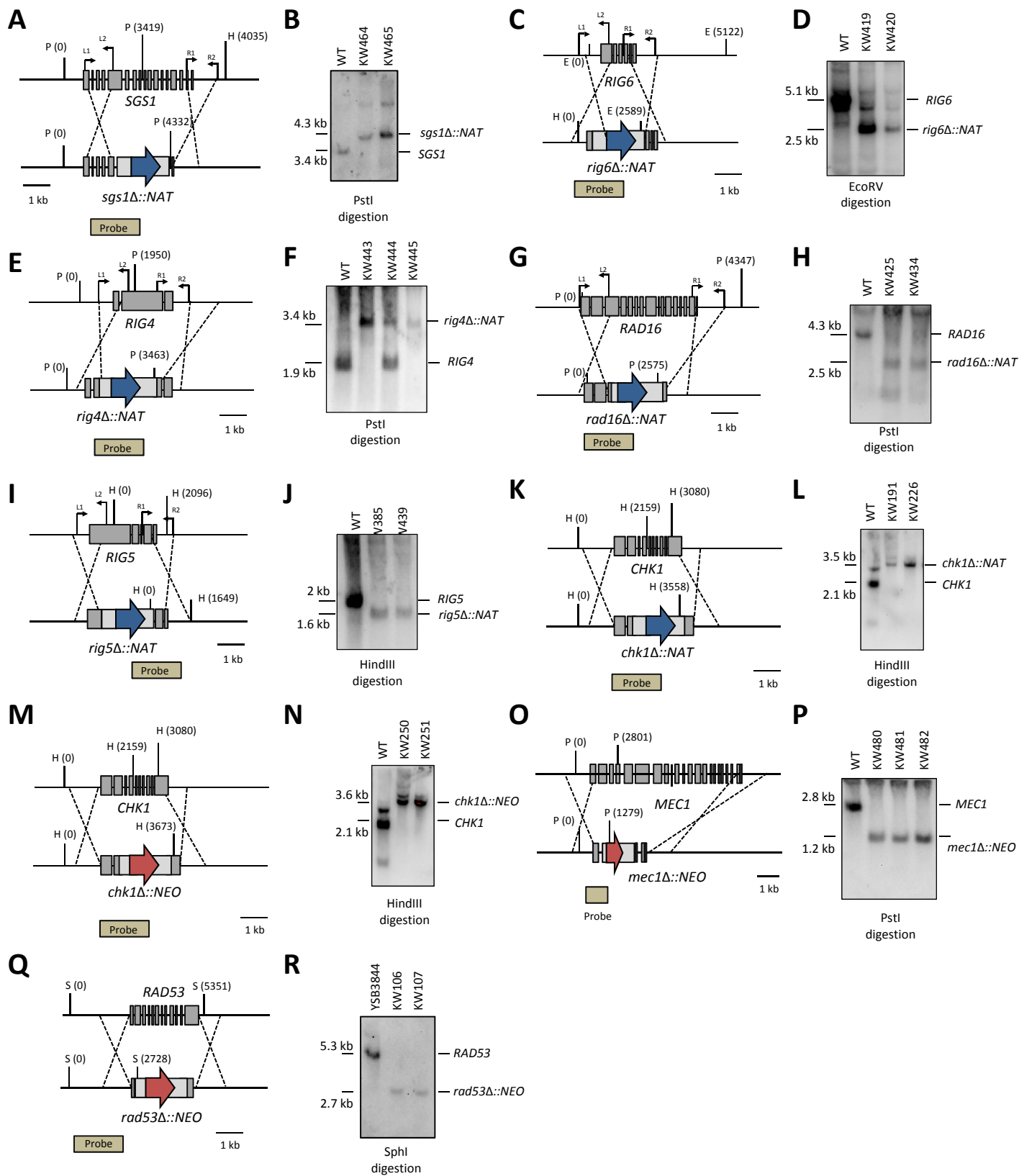

Supplement: FIG S2 [file mbo004184242sf2.pdf]

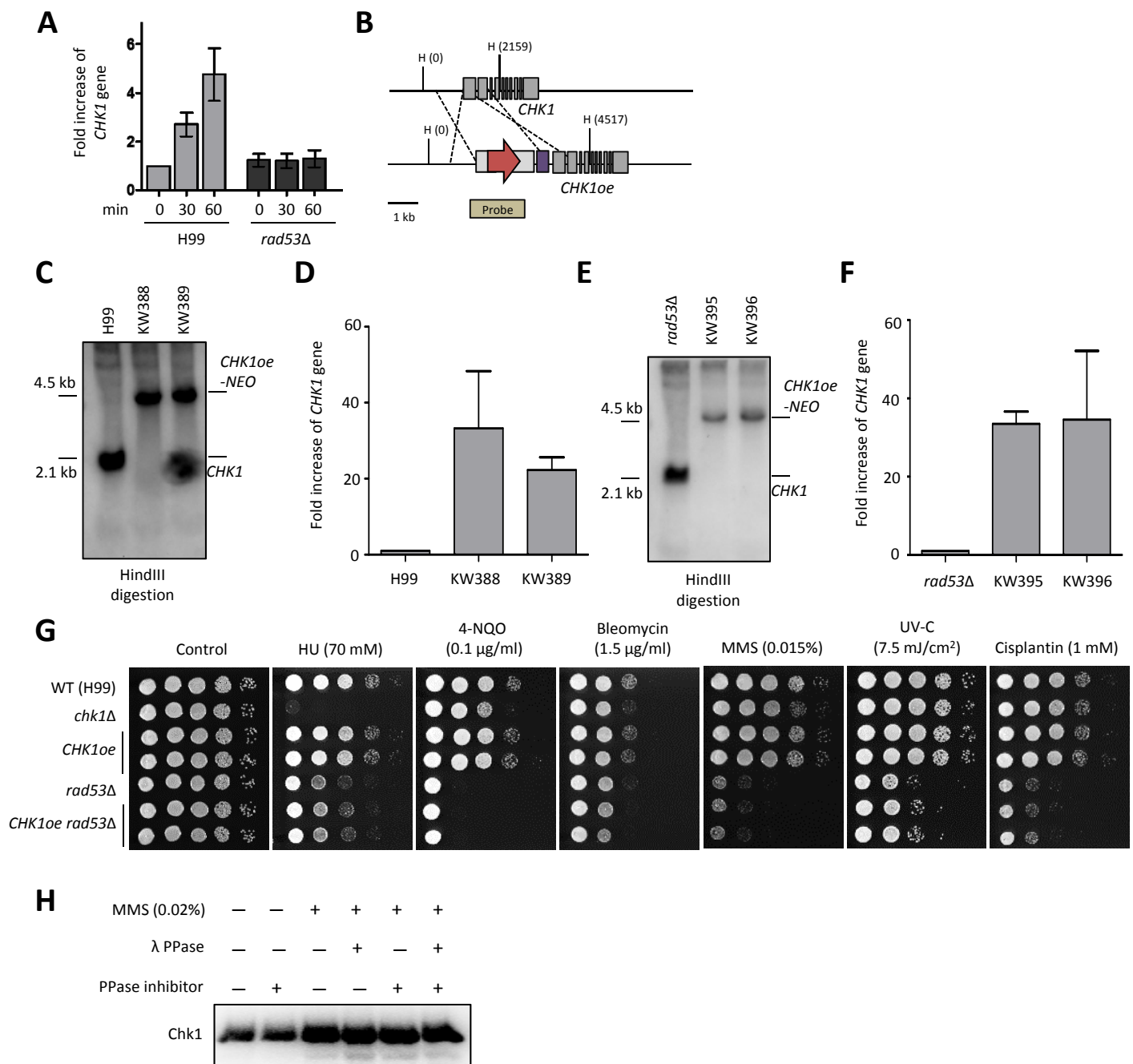

Supplement: FIG S3 [file mbo004184242sf3.pdf]

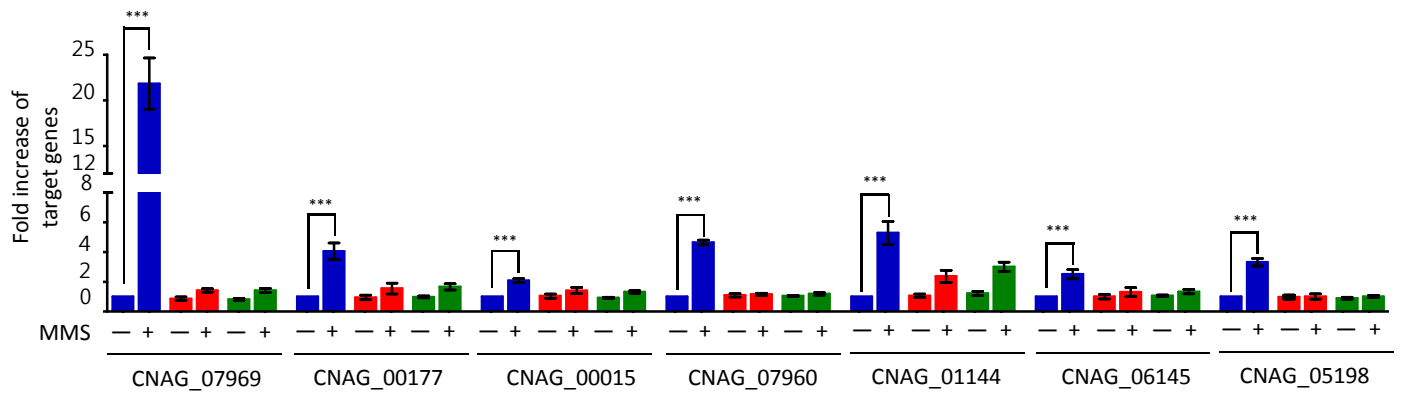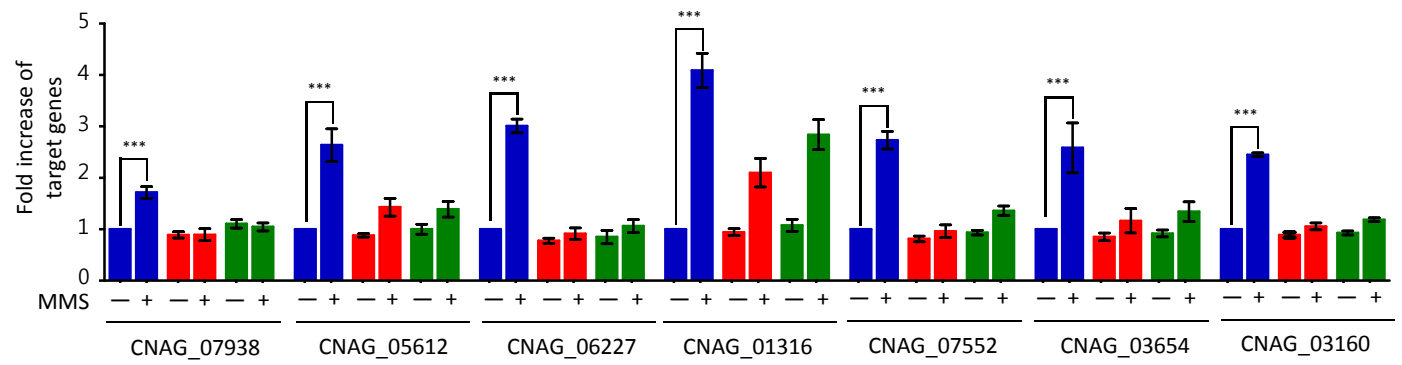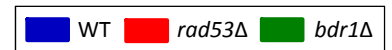

Supplement: FIG S4 [file mbo004184242sf4.pdf]

**A**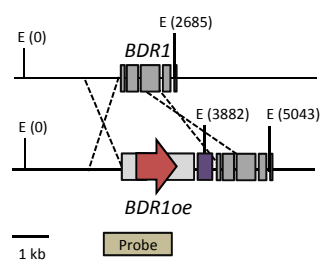**B**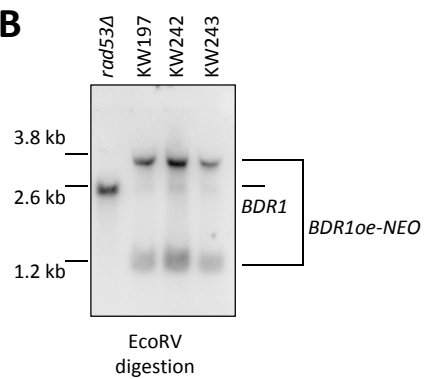**C**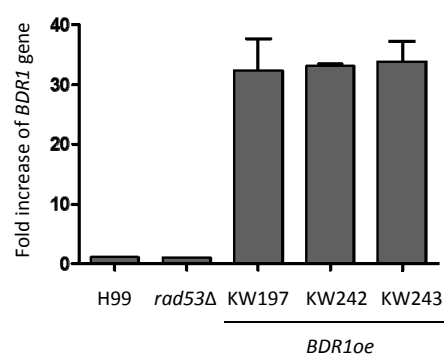

Supplement: FIG S5 [file mbo004184242sf5.pdf]

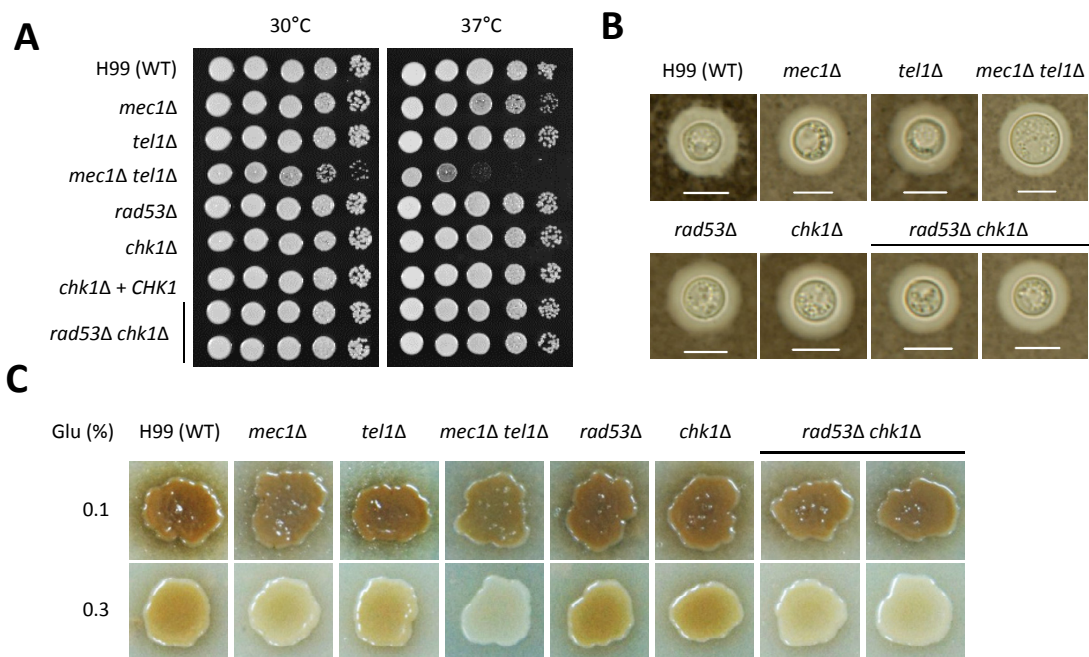

Supplement: FIG S6 [file mbo004184242sf6.pdf]
