# Supplementary material for: Rad53- and Chk1-Dependent DNA Damage Response Pathways Cooperatively Promote Fungal Pathogenesis and Modulate Antifungal Drug Susceptibility
Source: mBio. 2019 Jan 2;10(1):e01726-18. doi: 10.1128/mBio.01726-18 (PMC6315099; doi:10.1128/mBio.01726-18)
Supplement: TEXT S1 [file mbo004184242s1.docx]

**Text S1. Supplemental methods**

**Construction of *BDR1* or *CHK1* overexpression strains.** To construct constitutive gene overexpression strains, the native promoter of each gene was replaced with the histone H3 promoter using an amplified homologous recombination cassette. To generate *BDR1* overexpression strains, we generated *P_H3_:BDR1* replacement cassettes, as previously described ([1](#_ENREF_1)) and introduced them into *C. neoformans* by biolistic transformation. To construct the *CHK1* overexpression strain, B4329/J206 and J207/B4334 primer pairs were used for amplification of its 5’-flanking and 5’-coding regions, respectively, in the first round PCR. The *NEO-H3* promoter region was amplified using the B4017/B4018 primer pair. Next, the 5’-and 3’-regions of *P_H3_:CHK1* replacement cassettes were amplified by DJ-PCR using primer pairs B4329/B1887 and B4334/B1886, respectively. The *NEO*-marked H3 promoter replacement cassettes were introduced into the native promoter region of *CHK1* in the wild-type and *rad53*∆ (YSB3785) mutant strains (see Fig. S3C in the supplemental material). Stable transformants selected on YPD medium containing G418 were screened by diagnostic PCR. Next, the correct genotype of selected transformants was verified by Southern blot analysis, as previously described ([2](#_ENREF_2)). The constitutive overexpression of *BDR1* and *CHK1* was confirmed by qRT-PCR analysis using gene-specific primers, listed in Table S2.

**Construction of *RAD53* and *CHK1* complemented strains and Rad53-Gfp strain.** To confirm the phenotypes observed in *rad53*∆ and *chk1*∆ mutants, we constructed the corresponding complemented strains as follows: For the *rad53*∆+*RAD53* complemented strain, the genomic fragment of *RAD53* gene containing its promoter, ORF, and terminator was amplified by PCR using primers B6977 and B6978 (containing HindIII site) and H99 genomic DNA as the template. Next, the PCR-amplified gene product was cloned into the plasmid pTOP–V2 (Enzynomics) to produce pTOP–RAD53. After confirming the DNA sequence of the *RAD53* insert, it was sub-cloned into the pJAF12 plasmid containing NEO-selection marker to produce pJAF12-RAD53. The NsiI-digested linearized pJAF12-RAD53 was re-integrated into the native *RAD53* locus of the *rad53*∆ mutant (YSB3785) by biolistic transformation. The correct insertion of *RAD53* gene was confirmed by diagnostic PCR using primers B2572 and B2577. For the *chk1*∆+*CHK1* complemented strain, a genomic DNA fragment of the *CHK1* gene harboring its promoter, ORF, and terminator was amplified by PCR using primers B6993 and B6994 containing NotI site. Next, the PCR-amplified *CHK1* gene product was cloned into pGEM -T-Easy vector (Promega) to produce pGEM-CHK1. After confirming the DNA sequence, the *CHK1* insert was sub-cloned into the plasmid pJAF12 to produce pJAF12-CHK1. The MfeI-digested and linearized pJAF12-CHK1 was introduced into the native locus of *CHK1* in the *chk1*Δ mutant (KW191) by biolistic transformation. The correct insertion of *CHK1* gene was confirmed by diagnostic PCR. To elucidate the cellular localization of Rad53, we constructed *rad53*∆+*RAD53-GFP* strain as follows. The terminator region of *RAD53* (RAD53t) was PCR-amplified using primers J85 (containing SacII site) and J68. The GFP region was PCR-amplified using primers containing a glycine-serine linker with SacII and HindIII sites and pACT-HOG1fGFP (YSBE142) as the template. These PCR products were combined and used for overlap PCR with primers J84 and J85. Next, the PCR product was cloned into a pGEM-T-Easy vector (Promega) to produce pGEM-GFP-RAD53t and confirmed by DNA sequencing. The promoter-ORF region of *RAD53* (*RAD53PE*) was PCR-amplified using primers B6977 and J67 containing HindIII site with pTOP-RAD53 as the template, and then cloned into pGEM-T-Easy vector to produce pGEM-RAD53PE. After confirming the insert sequence, the HindIII-digested RAD53PE was sub-cloned into pGEM-GFP-RAD53t to construct the pGEM-RAD53PE-GFP-RAD53t plasmid. The SacII-digested RAD53PE-GFP-RAD53t was sub-cloned into pJAF12 to generate pJAF12-RAD53-GFP. The NsiI-digested and linearized pJAF12-RAD53-GFP was transformed into *rad53*∆ mutant (YSB3785) by biolistic transformation. Re-integration of the *RAD53*-*GFP* gene into its native locus was confirmed by diagnostic PCR using B2577 and B6986 primers.

**Construction of *RAD53*^KD^ (*RAD53*^K230N D341A^) strains.** The kinase-dead *RAD53* mutants (K230N and D341A) were constructed as follows: To substitute lysine 230 for asparagine in *RAD53*, pTOP-*RAD53*^K230N^ was amplified by PCR using AccuPrime Pfx DNA polymerase (Thermo Fisher Scientific) with J248 and J249 primers and pTOP-RAD53 as the template, and subsequently digested with DpnI at 37ºC for 3 h for removing pTOP-RAD53. The DpnI-digested reaction mixture was introduced into competent cells by transformation and positive clones were screened and confirmed by DNA sequencing. To replace aspartate 314 with alanine, pTOP-RAD53^K230N D341A^ was amplified by PCR using J250 and J251 primers and pTOP-*RAD53*^K230N^ as the template, digested with DpnI at 37ºC for 3 h for removing pTOP-RAD53^K230N^. The DpnI-digested reaction was introduced into competent cells by transformation and positive clones were screened and confirmed by DNA sequencing. The HindIII-digested *RAD53*^K230N D341A^ insert was sub-cloned into the plasmid pJAF12 to generate pJAF12-RAD53^KD^. To construct *RAD53^KD^* mutants, the NsiI-digested linearized pJAF12-RAD53^KD^ was introduced into *rad53*∆ mutant (YSB3785) by biolistic transformation. The targeted or ectopic integration of the *RAD53^KD^* allele into the *rad53*∆*::NAT* allele was confirmed by diagnostic PCR, and expression of the mutated *RAD53* gene was confirmed by qRT-PCR analysis using *RAD53*-specific primers J212 and J213.

**Construction of Rad53-4xFLAG, Chk1-4xFLAG, and Bdr1-4xFLAG strains.** We constructed the Rad53-4xFLAG strain as follows. In the first round PCR, three separate PCR products were generated. The 3’-exon region of *RAD53* was amplified using primers B6921 and B6922 and H99 genomic DNA as the template. The *4xFLAG-HOG1ter-NEO* fragment was amplified using primers B354 and B6567 and the plasmid pNEO-4xFLAGht as the template ([3](#_ENREF_3)). The 3’-flanking region of *RAD53* was amplified using primers B6923 and B2575 and the H99 genomic DNA as the template. In the second round PCR, a fusion fragment including the 3’-exon region of *RAD53* and 5’-split region of the *4xFLAG-HOG1ter-NEO* fragment was amplified using primers B6921 and B1886 and the combined first round PCR products as template. A fusion fragment containing the 3’-split region of the *4xFLAG-HOG1ter-NEO* fragment and the 3’-flanking region of *RAD53* gene was amplified using primers B2575 and B1887 and the combined first round PCR products as template. The two DJ-PCR products were mixed and introduced into the H99 strain by biolistic transformation. We constructed Chk1-4xFLAG strains as follows. First, we amplified the following three fragments by PCR. The 3’-exon region of *CHK1* was amplified using a primer pair J72/J73 and the H99 genomic DNA as a template. Primers B354 and B6567 were used to amplify the *4xFLAG-HOG1ter-NEO* fragment. The 3’-flanking region of *CHK1* gene was amplified using primer pair J74/B4332 and H99 genomic DNA as the template. We then amplified a fusion fragment containing the 3’-exon region of *CHK1* and 5’-split region of the *4xFLAG-HOG1ter-NEO* fragment using primers J72 and B1886, and a fusion fragment containing the 3’-split region of the *4xFLAG-HOG1ter-NEO* fragment and the 3’-flanking region of *CHK1* using primers B4332 and B1887 with the combined first round PCR fragments as template. The two split PCR products were mixed and introduced into the H99 strain by biolistic transformation. We constructed Bdr1-4xFLAG tagging strains as follows. The 3’-exon and flanking regions of *BDR1* and *4xFLAG-HOG1ter-NEO* fragments were amplified in the first round PCR. In the second round PCR, a fusion fragment containing the 5’-split region of the *4xFLAG-HOG1ter-NEO* fragment and the 3’-exon region of *BDR1,* and a fusion fragment containing the 3’-split region of the *4xFLAG-HOG1ter-NEO* fragment and the 3’-flanking region of *BDR1* were amplified using primer pairs J294/B1887 and J272/B1886, respectively, using the combined first round PCR products as template. The two split PCR products were mixed and introduced into the H99 strain by biolistic transformation. The targeted-integration of the *RAD53*-*4xFLAG*, *CHK1-4xFLAG*, and *BDR1-4xFLAG* alleles into the corresponding native C-terminal region of each locus was initially screened by diagnostic PCR and further verified using Southern blot analysis (see Fig. S1 in the supplemental material).

**Chemical sensitivity test and γ-radiation test.** Each *Cryptococcus* strain was cultured in liquid YPD medium for 16 h at 30°C and serially diluted (1 to 10^4^ dilutions) with sterilized dH_2_O. Three microliters of diluted cells were spotted onto a solid YPD medium containing the indicated concentration of the following chemicals: HU, MMS, bleomycin, cisplatin, and 4-nitroquionoline N-oxide (4-NQO) for genotoxic stress susceptibility test, and amphotericin B, fluconazole, and flucytosine for antifungal drug susceptibility test. For γ-radiation and UV-C resistance test, serially diluted cells (1 to 10^4^ dilutions) were spotted onto a solid YPD medium and then exposed to the indicated dose of γ-radiation or UV-C. The plates were further incubated at 30°C for 1-3 days and photographed daily.

**In vitro virulence factor production assay.** For capsule and melanin assays, *C. neoformans* cells were cultured overnight (16 h) at 30°C in a liquid YPD medium. For the melanin assay, 5 μl of cells was spotted on agar-based Niger seed and L-DOPA media, which contained the indicated concentration of glucose (0.1%, 0.2%, and 0.3%). The plates were incubated at 30°C and 37°C, monitored, and photographed daily. For the capsule assay, the cultured cells were spotted onto agar-based DMEM and further incubated at 37 °C. After incubation, cells were scrapped from the agar-based DMEM and resuspended in PBS stained with india ink (BACTIDROP^TM^; Remel, San Diego, CA). The stained cells were observed using Olympus BX51 microscope equipped with a SPOT Insight digital camera (Diagnostic Instruments Inc.). For quantitative measurement of capsule, the relative capsule diameter was determined by the following equation: (*D_w_*–*D_c_*) × *D_w_*, where *D_w_* and *D_c_* indicate the diameter of the whole cell body and the diameter of the cell body, respectively ([4](#_ENREF_4)).

**Total RNA extraction and quantitative RT-PCR.** To investigate downstream genes regulated by Rad53, total RNA was isolated from the wild type and *rad53*∆ mutant as follows: Strains were grown in 40 ml liquid YPD medium for 16 h at 30^o^C. Then, overnight culture was inoculated into 100 ml fresh YPD medium and adjusted to OD_600_=0.2. The cells were further incubated until OD_600_ reached approximately 0.6. Fifty milliliters of cells were pelleted for the zero-time sample and the remaining cells were exposed to γ-radiation (0.5 kGy). After γ-radiation exposure, cells were further incubated at 30^o^C for 30 min. Total RNA was isolated by the Trizol reagent (EasyBlue; Intron) as previously described ([5](#_ENREF_5)) and further purified using RNeasy spin column (Qiagen) according to the manufacturer’s protocol. To monitor relative expression of downstream genes controlled by Rad53, we conducted qRT-PCR analysis with gene-specific primers listed in Table S2 using CFX96 real-time PCR detection system (Bio-Rad). The cDNA was synthesized using PrimeScript^TM^ 1st strand cDNA synthesis kit (TAKARA) from total RNA extracted from the wild-type strain and *rad53*∆ mutant. Relative expression of the target genes was determined using 2^-∆∆Ct^ method, and statistical analyses were performed using one-way analysis of variance (ANOVA) with Bonferroni’s multiple-comparison test (GraphPad Software Inc.).

**Chromatin immunoprecipitation (ChIP) qPCR assay.** Cells harboring Bdr1-4xFLAG were cultured in liquid YPD medium for 16 h at 30^o^C. Next, cells were adjusted to OD_600_ of 0.2 in 100 ml of YPD and further incubated at 30^o^C until OD_600_ reached approximately 0.8. Fifty milliliters of cells were pelleted for the zero-time sample and the remaining cells were treated with MMS (0.02%) for 1 h. The procedures for cross-linking, cell lysis, and chromatin immunoprecipitation were carried out as previously described ([6](#_ENREF_6)).

**REFERENCES**

1. **Jung KW, Yang DH, Kim MK, Seo HS, Lim S, Bahn YS.** 2016. Unraveling Fungal Radiation Resistance Regulatory Networks through the Genome-Wide Transcriptome and Genetic Analyses of *Cryptococcus neoformans*. MBio **7**.

2. **Jung KW, Kim SY, Okagaki LH, Nielsen K, Bahn YS.** 2011. Ste50 adaptor protein governs sexual differentiation of *Cryptococcus neoformans* via the pheromone-response MAPK signaling pathway. Fungal Genet Biol **48:**154-165.

3. **So YS, Yang DH, Jung KW, Huh WK, Bahn YS.** 2017. Molecular Characterization of Adenylyl Cyclase Complex Proteins Using Versatile Protein-Tagging Plasmid Systems in *Cryptococcus neoformans*. J Microbiol Biotechnol **27:**357-364.

4. **Bahn YS, Hicks JK, Giles SS, Cox GM, Heitman J.** 2004. Adenylyl cyclase-associated protein Aca1 regulates virulence and differentiation of *Cryptococcus neoformans* via the cyclic AMP-protein kinase A cascade. Eukaryot Cell **3:**1476-1491.

5. **Ko YJ, Yu YM, Kim GB, Lee GW, Maeng PJ, Kim SS, Floyd A, Heitman J, Bahn YS.** 2009. Remodeling of global transcription patterns of *Cryptococcus neoformans* genes mediated by the stress-activated HOG signaling pathways. Eukaryot Cell **8:**1197-1217.

6. **Yang DH, Jung KW, Bang S, Lee JW, Song MH, Floyd-Averette A, Festa RA, Ianiri G, Idnurm A, Thiele DJ, Heitman J, Bahn YS.** 2017. Rewiring of Signaling Networks Modulating Thermotolerance in the Human Pathogen *Cryptococcus neoformans*. Genetics **205:**201-219.
