# Supplementary material for: Rad53- and Chk1-Dependent DNA Damage Response Pathways Cooperatively Promote Fungal Pathogenesis and Modulate Antifungal Drug Susceptibility
Source: mBio. 2019 Jan 2;10(1):e01726-18. doi: 10.1128/mBio.01726-18 (PMC6315099; doi:10.1128/mBio.01726-18)
Supplement: TABLE S2 [file mbo004184242st2.docx]

**TABLE S2. Strains used in this study**

| **Strain** | **Genotype** | **Parent** | **Reference** |
| --- | --- | --- | --- |
| H99 | *MAT*α |  | ([1](#_ENREF_1)) |
| YSB3611 | *MAT*α *CNAG_02233* (*MEC1*)*::NAT*#204 | H99 | ([2](#_ENREF_2)) |
| YSB3844 | *MAT*α *CNAG_05771* (*TEL1*)*::NAT*#225 | H99 | ([2](#_ENREF_2)) |
| YSB3785 | *MAT*α *CNAG_05216* (*RAD53*)*::NAT*#184 | H99 | ([2](#_ENREF_2)) |
| KW1 | *MAT*α *CNAG_05216* (*RAD53*)*::NAT* pJAF12-*CNAG_05216* (*RAD53*)-*NEO* | YSB3785 | This study |
| KW62 | *MAT*α *CNAG_05216* (*RAD53*)*::NAT* pJAF12-*CNAG_05216* (*RAD53*)*:GFP*-*NEO* | YSB3785 | This study |
| KW210 | *MAT*α *CNAG_05216* (*RAD53*)*::NAT* pJAF12-*CNAG_05216* (*RAD53*)*KD*-*NEO* | YSB3785 | This study |
| YSB3806 | *MAT*α *CNAG_05216* (*RAD53*)*-*4xFLAG-NEO | H99 | This study |
| YSB3807 | *MAT*α *CNAG_05216* (*RAD53*)*-*4xFLAG-NEO | H99 | This study |
| KW191 | *MAT*α *CNAG_03167* (*CHK1*)*::NAT*#234 | H99 | This study |
| KW226 | *MAT*α *CNAG_03167* (*CHK1*)*::NAT*#234 | H99 | This study |
| KW355 | *MAT*α *CNAG_03167* (*CHK1*)*::NAT*#234 pJAF12-CNAG_03167(*CHK1*)-NEO | KW191 | This study |
| KW250 | *MAT*α *CNAG_05216* (*RAD53*)*::NAT CNAG_03167* (*CHK1*)*::NEO* | YSB3785 | This study |
| KW251 | *MAT*α *CNAG_05216* (*RAD53*)*::NAT CNAG_03167* (*CHK1*)*::NEO* | YSB3785 | This study |
| KW69 | *MAT*α *CNAG_03167* (*CHK1*)*-*4xFLAG-NEO | H99 | This study |
| KW70 | *MAT*α *CNAG_03167* (*CHK1*)*-*4xFLAG-NEO | H99 | This study |
| KW480 | *MAT*α *CNAG_05771* (*TEL1*)*::NAT*#225 *CNAG_02233* (*MEC1*)*::NEO* | YSB3844 | This study |
| KW481 | *MAT*α *CNAG_05771* (*TEL1*)*::NAT*#225 *CNAG_02233* (*MEC1*)*::NEO* | YSB3844 | This study |
| KW482 | *MAT*α *CNAG_05771* (*TEL1*)*::NAT*#225 *CNAG_02233* (*MEC1*)*::NEO* | YSB3844 | This study |
| KW102 | *MAT*α *CNAG_05216* (*RAD53*)*-*4xFLAG-NEO *CNAG_02233* (*MEC1*)*::NAT* | YSB3806 | This study |
| KW103 | *MAT*α *CNAG_05216* (*RAD53*)*-*4xFLAG-NEO *CNAG_02233* (*MEC1*)*::NAT* | YSB3806 | This study |
| KW104 | *MAT*α *CNAG_05216* (*RAD53*)*-*4xFLAG-NEO *CNAG_05771* (*TEL1*)*::NAT* | YSB3806 | This study |
| KW105 | *MAT*α *CNAG_05216* (*RAD53*)*-*4xFLAG-NEO *CNAG_05771* (*TEL1*)*::NAT* | YSB3806 | This study |
| KW106 | *MAT*α *CNAG_05771* (*TEL1*)*::NAT*#225 *CNAG_05216* (*RAD53*)*::NEO* | YSB3844 | This study |
| KW107 | *MAT*α *CNAG_05771* (*TEL1*)*::NAT*#225 *CNAG_05216* (*RAD53*)*::NEO* | YSB3844 | This study |
| KW449 | *MAT*α *CNAG_05216* (*RAD53*)*-*4xFLAG-NEO *CNAG_05771* (*TEL1*)*::NAT CNAG_02233* (*MEC1*)*:: HYG* | KW104 | This study |
| KW450 | *MAT*α *CNAG_05216* (*RAD53*)*-*4xFLAG-NEO *CNAG_05771* (*TEL1*)*::NAT CNAG_02233* (*MEC1*)*:: HYG* | KW104 | This study |
| KW170 | *MAT*α *CNAG_03167* (*CHK1*)*-*4xFLAG-NEO *CNAG_02233* (*MEC1*)*::NAT* | KW69 | This study |
| KW161 | *MAT*α *CNAG_03167* (*CHK1*)*-*4xFLAG-NEO *CNAG_05771* (*TEL1*)*::NAT* | KW69 | This study |
| KW162 | *MAT*α *CNAG_03167* (*CHK1*)*-*4xFLAG-NEO *CNAG_05771* (*TEL1*)*::NAT* | KW69 | This study |
| KW408 | *MAT*α *CNAG_03167* (*CHK1*)*-*4xFLAG-NEO *CNAG_05216* (*RAD53*)*::NAT* | KW69 | This study |
| KW409 | *MAT*α *CNAG_03167* (*CHK1*)*-*4xFLAG-NEO *CNAG_05216* (*RAD53*)*::NAT* | KW69 | This study |
| KW410 | *MAT*α *CNAG_03167* (*CHK1*)*-*4xFLAG-NEO *CNAG_05216* (*RAD53*)*::NAT* | KW69 | This study |
| KW198 | *MAT*α *PH3:CNAG_02589* (*BDR1*)*-NEO* | H99 | ([3](#_ENREF_3)) |
| KW199 | *MAT*α *PH3:CNAG_02589* (*BDR1*)*-NEO* | H99 | ([3](#_ENREF_3)) |
| KW197 | *MAT*α *PH3:CNAG_02589* (*BDR1*)*-NEO CNAG_05216* (*RAD53*)*::NAT*#184 | YSB3785 | This study |
| KW242 | *MAT*α *PH3:CNAG_02589* (*BDR1*)*-NEO CNAG_05216* (*RAD53*)*::NAT*#184 | YSB3785 | This study |
| KW238 | *MAT*α *CNAG_02589* (*BDR1*)*::NAT CNAG_05216* (*RAD53*)*::NEO* | KW137 | This study |
| KW190 | *MAT*α *CNAG_05216* (*RAD53*)*::NAT CNAG_02589* (*BDR1*)*::NEO* | YSB3785 | This study |
| KW388 | *MAT*α *PH3:CNAG_03167* (*CHK1*)*-NEO* | H99 | This study |
| KW389 | *MAT*α *PH3:CNAG_03167* (*CHK1*)*-NEO* | H99 | This study |
| KW395 | *MAT*α *PH3:CNAG_03167* (*CHK1*)*-NEO CNAG_05216* (*RAD53*)*::NAT*#184 | YSB3785 | This study |
| KW396 | *MAT*α *PH3:CNAG_03167* (*CHK1*)*-NEO CNAG_05216* (*RAD53*)*::NAT*#184 | YSB3785 | This study |
| KW202 | *MAT*α *CNAG_02589* (*BDR1*)*-*4xFLAG-NEO | H99 | This study |
| KW443 | *MAT*α *CNAG_05341* (*RIG4*)*::NAT* | H99 | This study |
| KW445 | *MAT*α *CNAG_05341* (*RIG4*)*::NAT* | H99 | This study |
| KW385 | *MAT*α *CNAG_07564* (*RIG5*)*::NAT#43* | H99 | This study |
| KW439 | *MAT*α *CNAG_07564* (*RIG5*)*::NAT#43* | H99 | This study |
| KW419 | *MAT*α *CNAG_03906* (*RIG6*)*::NAT#56* | H99 | This study |
| KW420 | *MAT*α *CNAG_03906* (*RIG6*)*::NAT#56* | H99 | This study |
| KW425 | *MAT*α *CNAG_02512* (*RAD16*)*::NAT#56* | H99 | This study |
| KW434 | *MAT*α *CNAG_02512* (*RAD16*)*::NAT#56* | H99 | This study |
| KW464 | *MAT*α *CNAG_03654* (*SGS1*)*::NAT#56* | H99 | This study |
| KW465 | *MAT*α *CNAG_03654* (*SGS1*)*::NAT#56* | H99 | This study |

Each *NAT-STM#* indicates the Nat^r^ marker with a unique signature tag.

**References**

1. **Perfect JR, Ketabchi N, Cox GM, Ingram CW, Beiser CL.** 1993. Karyotyping of *Cryptococcus neoformans* as an epidemiological tool. J Clin Microbiol **31:**3305-3309.

2. **Lee KT, So YS, Yang DH, Jung KW, Choi J, Lee DG, Kwon H, Jang J, Wang LL, Cha S, Meyers GL, Jeong E, Jin JH, Lee Y, Hong J, Bang S, Ji JH, Park G, Byun HJ, Park S, Park YM, Adedoyin G, Kim T, Averette AK, Choi JS, Heitman J, Cheong E, Lee YH, Bahn YS.** 2016. Systematic fungal analysis of kinases in the fungal pathogen *Cryptococcus neoformans*. Nat Commun **7:**12766.

3. **Jung KW, Yang DH, Kim MK, Seo HS, Lim S, Bahn YS.** 2016. Unraveling Fungal Radiation Resistance Regulatory Networks through the Genome-Wide Transcriptome and Genetic Analyses of *Cryptococcus neoformans*. MBio **7**.
