# Supplementary material for: Rad53- and Chk1-Dependent DNA Damage Response Pathways Cooperatively Promote Fungal Pathogenesis and Modulate Antifungal Drug Susceptibility
Source: mBio. 2019 Jan 2;10(1):e01726-18. doi: 10.1128/mBio.01726-18 (PMC6315099; doi:10.1128/mBio.01726-18)
Supplement: TABLE S3 [file mbo004184242st3.docx]

**TABLE S3. Primers used in this study**

|  |  | | |  |  |  |
| --- | --- | --- | --- | --- | --- | --- |
| Primer Name | | Sequence (5’—3’) | Comment | | | |
| B79 | TGTGGATGCTGGCGGAGGATA | | Screening primer on ACT promoter | | | |
| B1026 | GTAAAACGACGGCCAGTGAGC | | M13 forward (extended) | | | |
| B1027 | CAGGAAACAGCTATGACCATG | | M13 reverse (extended) | | | |
| B1454 | AAGGTGTTCCCCGACGACGAATCG | | NSL-NAT | | | |
| B1455 | AACTCCGTCGCGAGCCCCATCAAC | | NSR-NAT | | | |
| B1886 | TGGAAGAGATGGATGTGC | | NSL-NEO | | | |
| B1887 | ATTGTCTGTTGTGCCCAG | | NSR-NEO | | | |
| B4017 | GCATGCAGGATTCGAGTG | | H3 promoter- left flanking primer 1 | | | |
| B4018 | GTGATAGATGTGTTGTGGTG | | H3 promoter- right flanking primer 2 | | | |
| B6567 | GCATGCGGCGCGCCAGAT | | 4xFLAG NEO marker – left flanking primer 1 | | | |
| B354 | GCATGCAGGATTCGAGTG | | 4xFLAG NEO marker – left flanking primer 1 | | | |
| J454 | TGCTCAAGTACGAACCGACC | | *RIG6*  (CNAG_03906) qRT primer 1 | | | |
| J455 | TCGGACCTGTTGATCCATTG | | *RIG6* (CNAG_03906) qRT primer 2 | | | |
| J558 | GCTATCTGGCTTACGCTCC | | *RIG4* (CNAG_05341) qRT primer 1 | | | |
| J559 | TTCCCAAGCCATCCCAAG | | *RIG4* (CNAG_05341) qRT primer 2 | | | |
| J503 | TCAAGTCGCTCGTGTTCTC | | *RAD16* (CNAG_02512) qRT primer 1 | | | |
| J504 | CACCTTCAAGTCGGCAAATG | | *RAD16* (CNAG_02512) qRT primer 2 | | | |
| J456 | GAAGGACTCTTCGGACATCG | | *RIG5* (CNAG_07564) qRT primer 1 | | | |
| J457 | TGGTGATACGCTTGTGTTCG | | *RIG5* (CNAG_07564) qRT primer 2 | | | |
| J570 | CTGTCTTCGGAACGTATTGC | | *SGS1* (CNAG_03654) qRT primer 1 | | | |
| J571 | CGACGCTTATTTGCCAACG | | *SGS1* (CNAG_03654) qRT primer 2 | | | |
| J212 | GGCTTTCTGAGCCCTCTTC | | *RAD53* qRT primer 1 | | | |
| J213 | ACTCGGGTTGTAATGAGTCG | | *RAD53* qRT primer 2 | | | |
| J560 | GAGCCGGAAAGAAGATTGC | | *RIG4* –5’ screening primer | | | |
| J561 | TTGCCGAACAGTCCTCTC | | *RIG4* – left flanking primer 1 | | | |
| J562 | TCACTGGCCGTCGTTTTACAAATTGACGCCATGCCTG | | *RIG4* – left flanking primer 2 | | | |
| J563 | CATGGTCATAGCTGTTTCCTGCCCATTACGCACCCTCTTC | | *RIG4* – right flanking primer 1 | | | |
| J564 | CGGTTTGCTCTCTTAGCCTC | | *RIG4* – right flanking primer 2 | | | |
| J565 | AACGGCGAAGAAGCTACC | | *RIG4* – probe primer for Southern blot | | | |
| J464 | ATTAGAACTCGCACCCGAAC | | *RIG5* –5’ screening primer | | | |
| J465 | TGATGGATGACACGCTCG | | *RIG5* – left flanking primer 1 | | | |
| J466 | TCACTGGCCGTCGTTTTACTACTATGCGAGGTGTGGGCTTC | | *RIG5* – left flanking primer 2 | | | |
| J467 | CATGGTCATAGCTGTTTCCTGAGAATACACTGGTGCCGAG | | *RIG5* – right flanking primer 1 | | | |
| J468 | TGGTTCGATGGTCTGAGAGG | | *RIG5* – right flanking primer 2 | | | |
| J584 | AACGCCCTTCAGTCTACC | | *RIG5* – probe primer for Southern blot | | | |
| J535 | CCTTTGCGAGCAAGATTGG | | *RIG6* –5’ screening primer | | | |
| J536 | AAACCCTGGTGCCAGTAC | | *RIG6* – left flanking primer 1 | | | |
| J537 | TCACTGGCCGTCGTTTTACTCCTTTGTGCTTTGCTGC | | *RIG6* – left flanking primer 2 | | | |
| J538 | CATGGTCATAGCTGTTTCCTGTGGATCAACAGGTCCGAAAG | | *RIG6* – right flanking primer 1 | | | |
| J539 | ACAACACCAACACATCTGC | | *RIG6* – right flanking primer 2 | | | |
| J540 | ACGCTTTCGGACCTGTTG | | *RIG6* – probe primer for Southern blot | | | |
| J517 | GTAATTCGGTGGCGGTTG | | *RAD16* –5’ screening primer | | | |
| J518 | TCGACGACGACACAAAGG | | *RAD16* – left flanking primer 1 | | | |
| J519 | TCACTGGCCGTCGTTTTACAAGCGGCACTTCAACCTC | | *RAD16* – left flanking primer 2 | | | |
| J520 | CATGGTCATAGCTGTTTCCTGGGTTCAACGTGAGTCCCAG | | *RAD16* – right flanking primer 1 | | | |
| J521 | CAACGCCACCTTCTTTCTC | | *RAD16* – right flanking primer 2 | | | |
| J522 | ATGGCTACGCACGTCTTG | | *RAD16* – probe primer for Southern blot | | | |
| J572 | CATTGTGCTGTGAGCAGG | | *SGS1* –5’ screening primer | | | |
| J573 | GGAGACACCCTCTGCATTG | | *SGS1* – left flanking primer 1 | | | |
| J574 | TCACTGGCCGTCGTTTTACTACTTCCTCGATACTGCCTCGTC | | *SGS1* – left flanking primer 2 | | | |
| J575 | CATGGTCATAGCTGTTTCCTGTTGCACGGGCGATAGAAC | | *SGS1* – right flanking primer 1 | | | |
| J576 | ACTCCTGAAGATGCAGTCC | | *SGS1* – right flanking primer 2 | | | |
| J577 | TGGCGTATGTCAGGATCTC | | *SGS1* – probe primer for Southern blot | | | |
| B4333 | ATACCACCACAAACGCCTC | | *CHK1* –5’ screening primer | | | |
| B4329 | GTATCTCCATCCCACACATC | | *CHK1* – left flanking primer 1 | | | |
| J305 | TCACTGGCCGTCGTTTTACAAATCTCCACCAACAGCCAG | | *CHK1* – left flanking primer 2 | | | |
| J306 | CATGGTCATAGCTGTTTCCTGCTTGGCAGACGCAATCTGG | | *CHK1* – right flanking primer 1 | | | |
| B4332 | CTGACAACAAGCAGCCTATC | | *CHK1* – right flanking primer 2 | | | |
| B4334 | GGACTACTTTCCGAAGGTTC | | *CHK1* – probe primer for Southern blot | | | |
| J351 | TTCAAGCGATTGGCCGAG | | *CHK1* – 5’ screening primer for FLAG tagging | | | |
| J72 | GGATGAACCTTCGGAAAG | | *CHK1* – left flanking primer 1 for FLAG tagging | | | |
| J73 | ATCTGGCGCGCCGCATGCCATGTCTCCTCGAATAACA | | *CHK1* – left flanking primer 2 for FLAG tagging | | | |
| J74 | CCACTCGAATCCTGCATGCATAAAGTCATGATGCAGTTT | | *CHK1* – right flanking primer 1 for FLAG tagging | | | |
| B4332 | CTGACAACAAGCAGCCTATC | | *CHK1* – right flanking primer 2 for FLAG tagging | | | |
| J75 | CTACTTTGGTTCCCGATACC | | *CHK1* – probe primer for Southern blot for FLAG tagging | | | |
| B6920 | GCCAGAGGTAAGTTTCATTCC | | *RAD53* –5’ screening primer for FLAG tagging | | | |
| B6921 | GCAGTATTTGGCTCCTGAG | | *RAD53* – left flanking primer 1 for FLAG tagging | | | |
| B6922 | ATCTGGCGCGCCGCATGCTTGAAGGCGCATAGATTTT | | *RAD53* – left flanking primer 2 for FLAG tagging | | | |
| B6923 | CCACTCGAATCCTGCATGCAGGCGTTTATCATTAGTAGA | | *RAD53* – right flanking primer 1 for FLAG tagging | | | |
| B2575 | GTAGACCCTCTTCTTCCTCG | | *RAD53* – right flanking primer 2 for FLAG tagging | | | |
| B6989 | ACCTGTTGGAGCAGCGAAAC | | *RAD53* – probe primer for Southern blot for FLAG tagging | | | |
| B6977 | CGCAAGCTTCCTTGGCTGACACTTTACC | | *RAD53* – primer 1 for complementation | | | |
| B6978 | CGCAAGCTTGTAGACCCTCTTCTTCCTCG | | *RAD53* – primer 2 for complementation | | | |
| B6986 | GGGAAAGCAGCCATTATTC | | *RAD53* – primer 1 for sequencing | | | |
| B6987 | TTGAAGAGCAGTAACGGAAC | | *RAD53* – primer 2 for sequencing | | | |
| B6988 | TGGAGTATGTAGATGGAGGTG | | *RAD53* – primer 3 for sequencing | | | |
| B6989 | ACCTGTTGGAGCAGCGAAAC | | *RAD53* – primer 4 for sequencing | | | |
| B6990 | CGCATCCATTATGAAGGAGTC | | *RAD53* – primer 5 for sequencing | | | |
| B2577 | ACCAATCAATCAGCCGAC | | *RAD53* – probe primer for Southern blot | | | |
| J67 | CGCAAGCTTTTGAAGGCGCATAGATTTTC | | *RAD53* GFP promoter exon | | | |
| J68 | GGACGAGCTGTACAAGTAAAGGCGTTTATCATTAGTAG | | *RAD53* terminator primer 1 | | | |
| J69 | GCGGCCGCGTAGACCCTCTTCTTCCTCG | | *RAD53* terminator primer 2 | | | |
| J70 | GCGGCCGCAAGCTTGGTGGCGGTGGCTCTGTGAGC | | Glycine serine linker (NotI+HindIII) GFP primer 1 | | | |
| J84 | CCGCGGAAGCTTGGTGGCGGTGGCTCTGTGAGC | | Glycine serine linker (SacII+HindIII) GFP primer 3 | | | |
| J85 | CCGCGGGTAGACCCTCTTCTTCCTCG | | *RAD53* teminator primer 3-SacII cut | | | |
| J248 | GGAATATGCGAGCCATCAATGTGAGAAAACACATGACAC | | *RAD53* point mutation for Lysine (Asparagine mutation) primer 1 | | | |
| J249 | GTGTCATGTGTTTTCTCACATTGATGGCTCGCATATTCC | | *RAD53* point mutation for Lysine (Asparagine mutation) primer 2 | | | |
| J250 | TCGAATAGTCAAAATTGCAGCTTTTGGCTTGGCTAAGATGA | | *RAD53* point mutation for Aspartic acid (Alanine mutation) primer 1 | | | |
| J251 | TCATCTTAGCCAAGCCAAAAGCTGCAATTTTGACTATTCGAG | | *RAD53* point mutation for Aspartic acid (Alanine mutation) primer 2 | | | |
| B6993 | CGGGCGGCCGCCATCCTTATCGCTTTTGGTC | | *CHK1* – primer 1 for complementation | | | |
| B6994 | CGGGCGGCCGCCTGACAACAAGCAGCCTATC | | *CHK1* – primer 2 for complementation | | | |
| B7008 | CTCTTCTCAAACTGGACCG | | *CHK1* – primer 1 for sequencing | | | |
| B7009 | ATCCAGGGCTACTACCTCAG | | *CHK1* – primer 2 for sequencing | | | |
| B7010 | AGGGCAAGACGAGACTACTG | | *CHK1* – primer 3 for sequencing | | | |
| B7011 | CGTCAAGAGGGTATGATGG | | *CHK1* – primer 4 for sequencing | | | |
| B7012 | TGTGGTGAGAGCGAATGAG | | *CHK1* – primer 5 for sequencing | | | |
| B2619 | CTGATTGAAGGAACTTACCTCG | | *MEC1* –5’ screening primer | | | |
| B2615 | TTCCTCATCCACGATACTTC | | *MEC1* – left flanking primer 1 | | | |
| B2616 | TCACTGGCCGTCGTTTTACGACAGAGGTTTGAGGATGC | | *MEC1* – left flanking primer 2 | | | |
| B2617 | CATGGTCATAGCTGTTTCCTGTTTTGTCCACGACCCTCTC | | *MEC1* – right flanking primer 1 | | | |
| B2618 | TCATTGCCACCTCCACCAAG | | *MEC1* – right flanking primer 2 | | | |
| B2620 | GGAGAAGTTCACGAAGGTCTG | | *MEC1* – probe primer for Southern blot | | | |
| B6881 | CATGGTCATAGCTGTTTCCTGCAGTATGGATGGGGAGTAATAG | | *TEL1* –5’ screening primer | | | |
| B6979 | ACCCTCCATACATCCTTCC | | *TEL1* – left flanking primer 1 | | | |
| B6980 | TCACTGGCCGTCGTTTTACGGCTATCGTTTCGGTAAGG | | *TEL1* – left flanking primer 2 | | | |
| B3418 | CATGGTCATAGCTGTTTCCTGGGTGTTACTGGGGTGAATG | | *TEL1* – right flanking primer 1 | | | |
| B3419 | CATCGCAGGCAACTATGAC | | *TEL1* – right flanking primer 2 | | | |
| B6996 | GAAATCGTCAAACTCGTTCC | | *TEL1* – probe primer for Southern blot | | | |
| J293 | GCAGCCGTTGATAATGCAC | | *BDR1* – 5’ screening primer for FLAG tagging | | | |
| J294 | TTGGCGGTGGAGATGATG | | *BDR1* – left flanking primer 1 for FLAG tagging | | | |
| J297 | ATCTGGCGCGCCGCATGCCCTGGCCTACATCAGTGTCT | | *BDR1* – left flanking primer 2 for FLAG tagging | | | |
| J298 | CCACTCGAATCCTGCATGCTGGCGTCCCACAACTTGGGA | | *BDR1* – right flanking primer 1 for FLAG tagging | | | |
| J272 | CATCAAACTCCTCAAACCC | | *BDR1* – right flanking primer 2 for FLAG tagging | | | |
| J303 | AGGAGAGATGGACATTGACAAC | | *BDR1* – probe primer for Southern blot for FLAG tagging | | | |
| J1 | TGGTGGTGAGGGAAAATG | | *RAD51* qRT primer 1 | | | |
| J2 | GCACCTCTTCACCATCAAG | | *RAD51* qRT primer 2 | | | |
| J3 | AAAGCGATGAGGATGACC | | *RDH54* qRT primer 1 | | | |
| J4 | CTCGTCTTTTTCAGCACG | | *RDH54* qRT primer 2 | | | |
| J5 | GCAAAATCTTTCAGCGTGTG | | *RAD54* qRT primer 1 | | | |
| J6 | CACAAAGTTTCGGGGTTG | | *RAD54* qRT primer 2 | | | |
| J220 | TAACACTCAGCGCCATCC | | *RIG1* qRT primer 1 | | | |
| J221 | CACATCGGCGTTCATCTCG | | *RIG1* qRT primer 2 | | | |
| J198 | ATTCGGGTGTAAAGCCTG | | *RIG2* qRT primer 1 | | | |
| J199 | AGAGGCTGGAATAGACGAGG | | *RIG2* qRT primer 2 | | | |
| J218 | AGACTGATCGCCAAGGTG | | *RIG3* qRT primer 1 | | | |
| J219 | ATGTGCATGTCCCGAGGTAG | | *RIG3* qRT primer 2 | | | |
| J208 | ACTCGTTCTCTGGTTCTCAC | | *CHK1* qRT primer 1 | | | |
| J209 | TCGCTAGGCAAGACAACG | | *CHK1* qRT primer 2 | | | |
| B679 | CGCCCTTGCTCCTTCTTCTATG | | *ACT1* qRT primer 1 | | | |
| B680 | GACTCGTCGTATTCGCTCTTCG | | *ACT1* qRT primer 2 | | | |
| J452 | ATCTGGTGCTCTCTTTCTCC | | CNAG_07969 qRT primer 1 | | | |
| J453 | AGCGCGAGAATGACGTAG | | CNAG_07969 qRT primer 2 | | | |
| J460 | TGTATATCGAAGCCGCGAC | | CNAG_00177 qRT primer 1 | | | |
| J461 | CCGTTTCCTTCCCGAAGTG | | CNAG_00177 qRT primer 2 | | | |
| J462 | TCAGCCACTCACACTTCG | | CNAG_00015 qRT primer 1 | | | |
| J463 | AGAAGGAGCAGGGAAAGC | | CNAG_00015 qRT primer 2 | | | |
| J458 | TGCTTCCATCCGTCATCG | | CNAG_07960 qRT primer 1 | | | |
| J459 | TCCTTTGCCTCACCGAAC | | CNAG_07960 qRT primer 2 | | | |
| J166 | TACACTACTGCGTCTGTCG | | CNAG_01144 qRT primer 1 | | | |
| J167 | TTCTCTGACATACCGAGCTG | | CNAG_01144 qRT primer 2 | | | |
| J505 | CGTCGTCGATGAACGTACTC | | CNAG_06145 qRT primer 1 | | | |
| J506 | CCTTGCGAGCTTCTAGCTG | | CNAG_06145 qRT primer 2 | | | |
| J192 | TGATTTGACCGACGCCATTC | | CNAG_05198 qRT primer 1 | | | |
| J193 | GACGCCTTGGTCAGTAAGC | | CNAG_05198 qRT primer 2 | | | |
| J962 | CCTGAGGAAGAGAAGCAGTTG | | CNAG_07938 qRT primer 1 | | | |
| J963 | GGCGAAATGAAGGTTGTCC | | CNAG_07938 qRT primer 2 | | | |
| J507 | CTGTACCAAATGCCGGACC | | CNAG_05612 qRT primer 1 | | | |
| J508 | GCGGGCTTTAGCTTTCTG | | CNAG_05612 qRT primer 2 | | | |
| J509 | GATGTGCTGCTTTCATCAGG | | CNAG_06227 qRT primer 1 | | | |
| J510 | TTTGCGCTTCTTCTCCAAGG | | CNAG_06227 qRT primer 2 | | | |
| J168 | TGACTACAATGCCGGAGC | | CNAG_01316 qRT primer 1 | | | |
| J169 | CCTTCGCTCGACATAACTTCC | | CNAG_01316 qRT primer 2 | | | |
| J568 | AAGCCATCCACCAATCCAG | | CNAG_07552 qRT primer 1 | | | |
| J569 | CATCGTTGAGAAGCGGCTG | | CNAG_07552 qRT primer 2 | | | |
| J570 | CTGTCTTCGGAACGTATTGC | | CNAG_03654 qRT primer 1 | | | |
| J571 | CGACGCTTATTTGCCAACG | | CNAG_03654 qRT primer 2 | | | |
| J11 | ACTGAAGCCCAAGTCCACC | | CNAG_03160 qRT primer 1 | | | |
| J12 | CCTAAGACTCGGTCGAAATGG | | CNAG_03160 qRT primer 2 | | | |
| YS3 | GAGCGCCCTCCGCGTCCGCTTC | | *RAD51* ChIP-qRT primer 1 | | | |
| YS4 | GTTCCTGCGAGGTGTTGCTGC | | *RAD51* ChIP-qRT primer 2 | | | |
| J877 | TGAGTGAAAGTGGCTCATCG | | *Β-tubulin* ChIP-qRT primer 1 | | | |
| J878 | AGCAAGCCAAAAACAACACC | | *Β-tubulin* ChIP-qRT primer 2 | | | |
| J879 | GCACCATACCTTCTACAATGAGC | | *ACT1* ChIP-qRT primer 1 | | | |
| J880 | CAGGAAGCTCGTAAGACTTTTCA | | *ACT1* ChIP-qRT primer 2 | | | |
